# Immunopathogenic and clinical implications of advanced tissue analysis in non-tuberculous mycobacterial infections in children

**DOI:** 10.3389/fimmu.2025.1597074

**Published:** 2025-06-26

**Authors:** Maximilian Seidl, Elham Bavafaye Haghighi, Anne Kathrin Lösslein, Markus Hufnagel, Florens Lohrmann, Christian Schneider, Daniela S. Kohlfürst, Werner Zenz, Gregor Gorkiewicz, Cornelia Feiterna-Sperling, Renate Krüger, Peter Bronsert, Christina Neppl, Kim Zoe Sommer, Verena Stehl, Melanie Boerries, Martin Kuntz, Philipp Henneke

**Affiliations:** ^1^ Institute for Immunodeficiency, Center for Chronic Immunodeficiency, Medical Center – University of Freiburg/Medical Faculty – University of Freiburg, Freiburg, Germany; ^2^ Center for Pathology, Medical Center – University of Freiburg/Medical Faculty – University of Freiburg, Freiburg, Germany; ^3^ Institute of Pathology, Heinrich Heine University and University Hospital of Duesseldorf, Duesseldorf, Germany; ^4^ Department of Tissue Dynamics and Regeneration, Max Planck Institute for Multidisciplinary Sciences, Goettingen, Germany; ^5^ Institute of Medical Bioinformatics and Systems Medicine, Medical Center – University of Freiburg/Medical Faculty – University of Freiburg, Freiburg, Germany; ^6^ Institute for Microbiology and Hygiene, Medical Center – University of Freiburg/Medical Faculty – University of Freiburg, Freiburg, Germany; ^7^ Institute for Infection Prevention and Control, Medical Center – University of Freiburg/Medical Faculty – University of Freiburg, Freiburg, Germany; ^8^ Department of General Pediatrics, Adolescent Medicine and Neonatology, Medical Center – University of Freiburg/Medical Faculty – University of Freiburg, Freiburg, Germany; ^9^ Department of Pediatrics and Adolescent Medicine, Division Pediatric Hemato-Oncology, Medical University of Graz, Graz, Austria; ^10^ Department of Paediatrics and Adolescent Medicine, Division of General Paediatrics, Medical University of Graz, Graz, Austria; ^11^ Diagnostic and Research Institute of Pathology, Medical University of Graz, Graz, Austria; ^12^ Department of Pediatric Respiratory Medicine, Immunology and Critical Care Medicine, Charité – Universitätsmedizin Berlin, corporate member of Freie Universität Berlin and Humboldt-Universität zu Berlin, Berlin, Germany; ^13^ German Cancer Consortium (DKTK), Partner site Freiburg, a partnership between DKFZ and Medical Center - University of Freiburg, Freiburg, Germany; ^14^ CIBSS – Center for Integrative Biological Signalling Studies, University of Freiburg, Freiburg, Germany

**Keywords:** non-tuberculous mycobacteria, lymphadenitis, granuloma, giant cells, children, complications, histology

## Abstract

**Objectives:**

Infections with non-tuberculous mycobacteria (NTM) in children usually affect the lymph nodes and surrounding tissue. Although the infection is typically self-limiting, it carries a substantial risk of complications due to persistent inflammation and invasive therapeutic interventions. Yet, the immunopathogenesis of the disease is obscure, as are biomarkers guiding treatment decisions.

**Methods:**

In this observational study, we analyzed histological samples collected in the NTMkids study to identify parameters associated with impaired wound healing and complicated disease progression. Samples from 33 patients (median age at first presentation 33 months) were investigated, with two consecutive biopsies in 9 patients.

**Results:**

Germinal centers, a scattered distribution of granuloma associated CD4+ T-cells, higher CD8+ T-cell density inside the necrosis and foamy epitheloid cells were associated with a favorable outcome. Tissue damage presenting clinically as liquefaction was associated with an adverse outcome.

**Conclusions:**

The identified tissue reaction patterns in NTM infections provide insights into the biology of NTM lymphadenitis in children and may aid in more precise treatment decisions.

## Introduction

Non-tuberculous mycobacteria (NTM) are ubiquitous in the environment, especially in water (1–3). Thus, exposure is frequent, yet usually remains without consequences. However, mycobacterial infections can cause prolonged disease courses driven by chronic inflammation (1, 4–6). A unique NTM infection entity, which almost exclusively affects otherwise healthy preschool children, is cervical lymphadenitis. Many cases resolve spontaneously, yet the median time to resolution is 40 weeks. In addition, differential diagnosis is challenging, and recurrence and scarring are frequent complications (7). Accordingly, although NTM are of relatively low virulence, they exhibit high tenacity leading to persistence over months to years. This is largely due to a predominantly intracellular survival in macrophages, linked to the formation of a heterocellular tissue structure called granuloma (5, 7). In granulomas, the production of antimycobacterial substances like reactive oxygen species (ROS) and nitric oxide (NO) go hand in hand with cellular transformation processes, including those inducing multinuclear giant cells (MGC) (8). MGC in turn are relatively permissive for mycobacteria and may thus be sites of mycobacterial latency (9). Thus, granulomas appear to have ambiguous roles. On the one hand, they locally restrict mycobacteria, which cannot be immediately killed, and thereby prevent dissemination. On the other hand, they represent sites of pathogen persistence and a slowly progressing infection as indicated by the associated tissue damage.

In the context of pulmonary tuberculosis, histopathologic characteristics have been exploited to categorize mycobacterial granuloma types using different classification systems (10). However, beyond the finding that large necrosis is associated with increased transmission rates, the granuloma type has found to be of little prognostic value (11). Histopathological analyses of NTM infections have focused on adult lung disease (12), whereas associations between granuloma histology and clinical course in pediatric infections have not been established prior to this study.

Consensus of how to best treat NTM lymphadenitis in children has not been reached. Whereas many specialists agree that early lymph node extirpation is advantageous for both diagnostic and therapeutic purposes, decision algorithms based on the integration of clinical and pathological information to guide how to proceed after extirpation, e.g., wait-and-watch, antibiotic therapy or secondary and more complete surgery, have not been established. This is largely due to the lack of reliable prognostic markers for the disease.

To break new ground in this area, we combined multiparameter histopathological analysis of surgical specimens available from the NTMkids study with comprehensive clinical data. This enabled us to identify several specific histopathological features, including maintained lymphoid structures, distribution patterns of granuloma associated CD4+ and CD8+ T-cells and foamy epitheloid cells to be indicative for a balanced immune reaction and an uncomplicated disease course.

## Methods

### Patients and samples

It was attempted to include all patients enrolled into the NTMkids study (13), who underwent surgical interventions, into the present analysis. We contacted all study centers or their respective pathological institutes asking to provide us with biopsy material of the patients. For 33 out of 138 patients, we were able to obtain material in sufficient quality and quantity allowing further analysis. For all other patients, no material of suitable quality for further analysis was available at the study centers. Clinical data was available from the NTMkids study. Outcomes were good vs impaired wound healing and complicated vs uncomplicated course. For the purpose of the study, wound healing was related to surgical intervention, “complicated course” was defined as illness lasting >12 months, >1 surgical intervention at the same site, or occurrence of major complications, such as substantial scarring or facial nerve palsy.

### Ethics

The present study was covered by consent and ethical approval of the original NTMkids study. Parental informed consent had been obtained for all children included in this study. Each participating center’s ethics committee granted ethics approval. The institutional review board of the Medical Center – University of Freiburg, Freiburg, Germany, was the lead approval agency under IRB no. 232/10.

### Histology

From all available samples, histological sections of 2-3 µm were taken and stained with hematoxylin and eosin (H&E), Elastica van Gieson (EVG), Ziehl-Neelsen (ZN) and immunohistochemistry for BCL2, BCL6, CD4, CD8, p53 and nitrotyrosine. All histological analyses were performed in a strictly blinded manner for the clinical data, outcomes and timepoints of sample acquisition from digitized slides. Histological analyses comprised qualitative and quantitative items. Where appropriate, software-aided analyses were performed. Details are provided in the supplement.

### Statistical evaluation

Due to the complexity and heterogeneity of the clinical course and of the histopathological features involved, we aimed to substantiate relevant associations by identifying a combination of classifiers to improve risk group categorization. Briefly, the prognostic impact of single variables to each outcome was analyzed by the Wilcoxon-Mann-Whitney test and correlation analysis. After identifying relevant variables and similar to the approach described by Saccenti et al. (14), the variables selected in the previous step were analyzed together with Binomial Logistic Regression using an ensemble of binary classifiers (15) by machine-learning. Details are provided in the supplement.

For patients with two biopsies from different time points, histological parameters from the first and second sample were compared by the paired Wilcoxon signed-rank test. Correlations between different histological parameters were analyzed with a pairwise correlation coefficient analysis (Spearman rho). A filter was applied, only giving the correlation coefficients > 0.3, with n > 3 and p < 0.05.

## Results

### Patients and samples

33 patients, 22 female and 11 male, were included into the study: 20 from Graz, Austria; 11 from Freiburg, Germany; 1 from Berlin, Germany; 1 from Leipzig, Germany; comprising 42 specimens: 33 samples from the first or sole surgical procedure, 9 from a second surgical procedure. In 19 patients, *M. avium-intracellulare* complex was identified, in 5 patients, other NTM species were found (2 *M. haemophilum*, 1 *M. celatum*, 1 *M. kansasii*, 1 *M. bohemicum*), and in 4 patients, NTM species could not be specified. It is noteworthy that acid-fast bacilli were not detectable microscopically by Ziehl-Neelsen staining in any of the samples. In 30 patients, the samples were from the head/neck region (91%). From 9 patients, samples of two timepoints were available: 7 from Graz, 2 from Freiburg. Median age at first presentation was 33 months, range 13 to 129 months. Outcomes were good vs impaired wound healing and complicated vs uncomplicated course. Wound healing was related to surgical intervention, “complicated course” was defined as illness lasting > 12 months, > 1 surgical intervention at the same site, or occurrence of major complications, such as substantial scarring or facial nerve palsy (reported by local study physician). 18 patients showed good wound healing (GWH) after surgery, and 15 impaired wound healing (IWH). 24 patients presented a complicated course of the disease, 9 an uncomplicated course. Median time to resolution of symptoms was 13 months. Based on multiparameter analysis, the patients analyzed here showed a higher frequency of facial nerve palsy, and a trend towards calcifications of the affected lymph node, without further significant differences to the original NTMkids study (**Supplementary Figure 1**, **Supplementary Table 3**).

**Figure 1:**
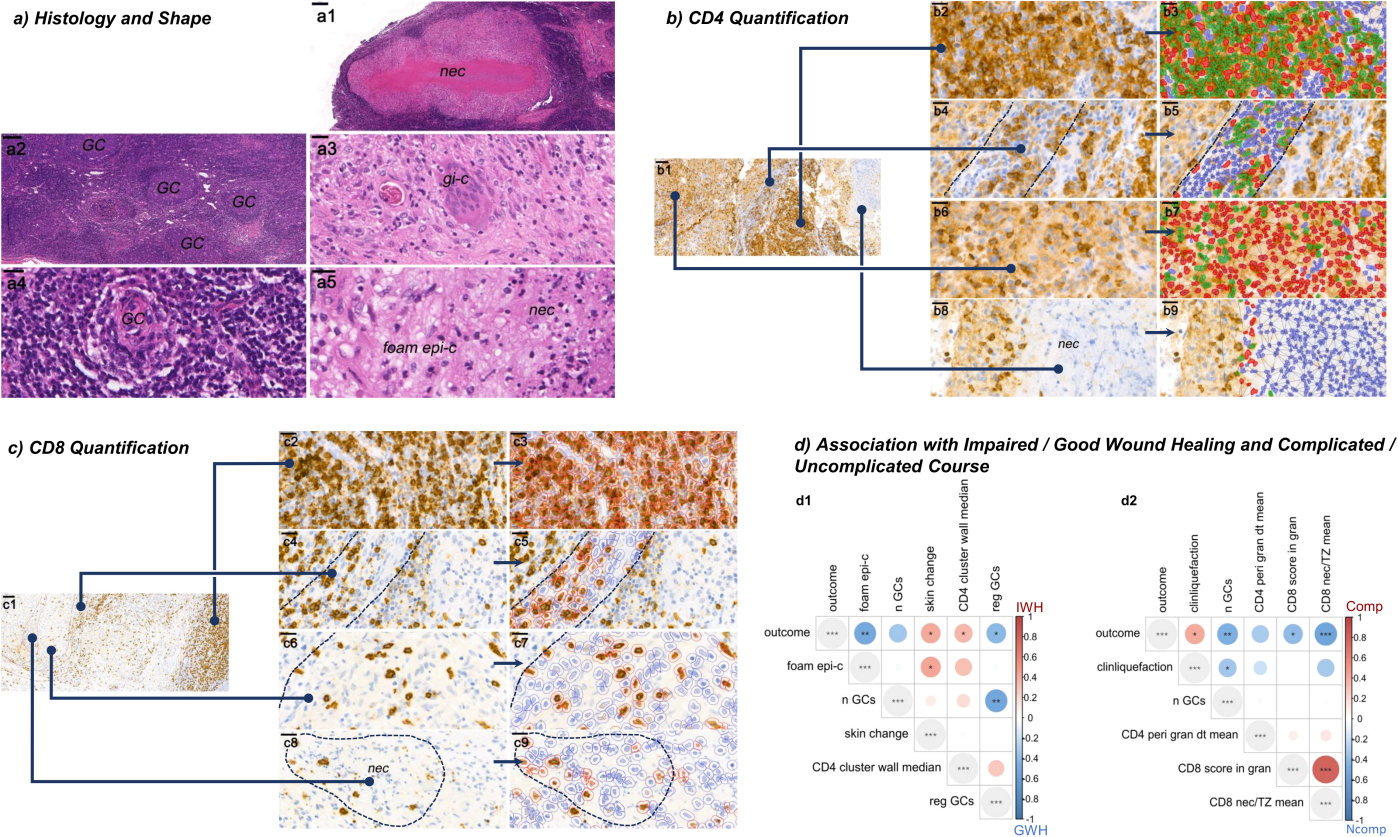
Granuloma histopathology and quantifications. **(a)** Granuloma histology and shape (H&E staining). **(a1)** Granuloma with central necrosis (nec). **(a2)** Frequent germinal centers (GC) in intact lymphoid tissue. **(a3)** Giant cell (gi-c). **(a4)** Small, regressive GC. **(a5)** Foamy epithelioid cells (foam epi-c). **(b)** CD4 quantification. CD4 positive cells (lymphocytes and macrophages) are stained in brown and quantified in different microanatomic structures: **(b1)** exemplary overview, blue lines pointing to higher magnifications: **(b2)** T-zone, **(b3)** the corresponding automated cell detection with CD4 T-cells (green), macrophages (red) and their distances (lines between the centroids of the cells). All cells connected with each other by lines belong to a cluster. **(b4)** Perigranulomatous area (80µm zone around the granuloma), **(b5)** corresponding quantification. **(b6)** Granuloma wall, **(b7)** corresponding quantification. **(b8)** Granuloma necrosis, **(b9)** corresponding quantification. **(c)** CD8 quantification. CD8 positive T-cells are stained in brown. **(c1)** exemplary overview similar to **(b1)**. **(c2)** T-zone, **(c3)** corresponding quantification (detected CD8 T-cells in red, negative cells in blue). Same for the perigranulomatous area **(c4)** and its quantification **(c5)**, the granuloma wall **(c6, c7)** and the granuloma necrosis **(c8, c9)**. **(d)** Association with wound healing **(d1)** and course **(d2)**, displayed as heatmaps of correlation coefficients. *, **, and *** for unadjusted p-values < 0.05, 0.01, and 0.001, accordingly. Visualizations: **(a1-5)** cases 11, 14, 19, 14, 14; magnifications indicated by bars and 200µm, 200µm, 50µm, 20µm, 20µm, respectively. **(b)** case 20, **(c)** case 6; magnifications indicated by bars for **(b1, c1)** 100µm and for **(b2-9, c2-9)** 20µm.

Overall, 37/42 specimens displayed necrotizing granulomas, 29/33 patients in the first or sole sample, 6/9 patients in the second sample. 23/42 specimens showed caseous necrosis, 8/42 fibrinoid necrosis and 15/42 suppurative necrosis. 11/42 specimens displayed more than one necrotic pattern. **Figure 1a** provides an overview of granuloma, epitheloid cell and giant cell patterns in the H&E staining. Further immunohistological analyses comprised quantifications of CD4+ and CD8+ T-cell distributions in the perigranulomatous area, the granuloma wall and the granuloma necrosis as depicted in **Figures 1b, c**, which was done using QuPath version 0.5.0 (16).

### Tissue damage, foamy epithelioid cells and infiltration patterns of CD4+ and CD8+ T-cells are associated with wound healing and the course of the disease

We previously identified clinical risk factors for a complicated vs. an uncomplicated course of disease (13). Concerning the histopathologic features, we identified a combination of classifiers for the risk group categorization “wound healing” and “course”.

**Figure 2:**
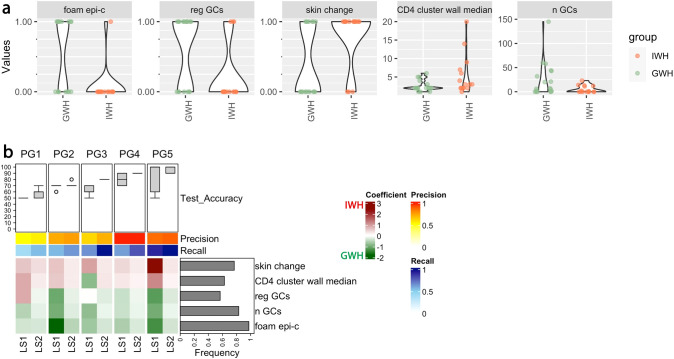
Patterns of epitheloid cells, germinal centers and CD4 infiltration are associated with the quality of wound healing. **(a)** Pairwise Analysis of depicted variables associated with “good wound healing” (GWH, n = 18) and “impaired wound healing” (IWH, n = 15). Unadjusted p-values (Wilcoxon-Mann-Whitney test) for comparisons: foam epi-c = 0.004, reg GCs = 0.02, skin change = 0.02, CD4 cluster wall median = 0.055, and n GCs = 0.06. Correlation coefficients: foam epi-c = -0.52, reg GCs = -0.41, skin change = 0.41, CD4 cluster wall median = 0.36, and n GCs = -0.34. **(b)** Binomial logistic regression analysis with classifiers categorizing IWH and GWH for different partition groups (PGs). Box plots: test accuracies of 50 models trained using k-fold cross validation (k=3) for each PG and learning step (LS) and evaluated based on the test data. 70% of the samples (n = 23) together with the augmented ones (n = 2) were used for training and 30% of the samples (n = 10) were used for testing. The mean of the precision and recall of the models calculated based on the test data is shown as two horizontal color bars. Bar plot, right side: Frequency of biomarker selection. The heatmap shows averages of the coefficients of the models. The green values indicate a positive, the red values a negative prognostic effect on the outcome "wound healing". foam epi-c = Foamy epitheloid cells; reg GCs = regressive germinal centers; skin change = skin change; CD4 cluster wall median = median number of CD4+ T-cells forming a cluster inside the granuloma wall; n GCs = Number of germinal centers.

Classifiers categorizing IWH (impaired wound healing) and GWH (good wound healing) for different partition groups (PGs) are displayed in **Figure 2**: Among the relevant variables, foamy epithelioid cells (foam epi-c) regressive germinal centers (reg GCs) and higher numbers of germinal centers (n GCs) were all independently associated with good wound healing (**Figures 1a2, a4, a5, d1**, **Figure 2** for the underlying classification). The phenotypes “skin change” and “CD4+ T-cell cluster size” in the granuloma wall (**Figures 1b1, b6, b7, d1**, **Figure 2** for the underlying classification) were associated with impaired wound healing.

**Figure 3:**
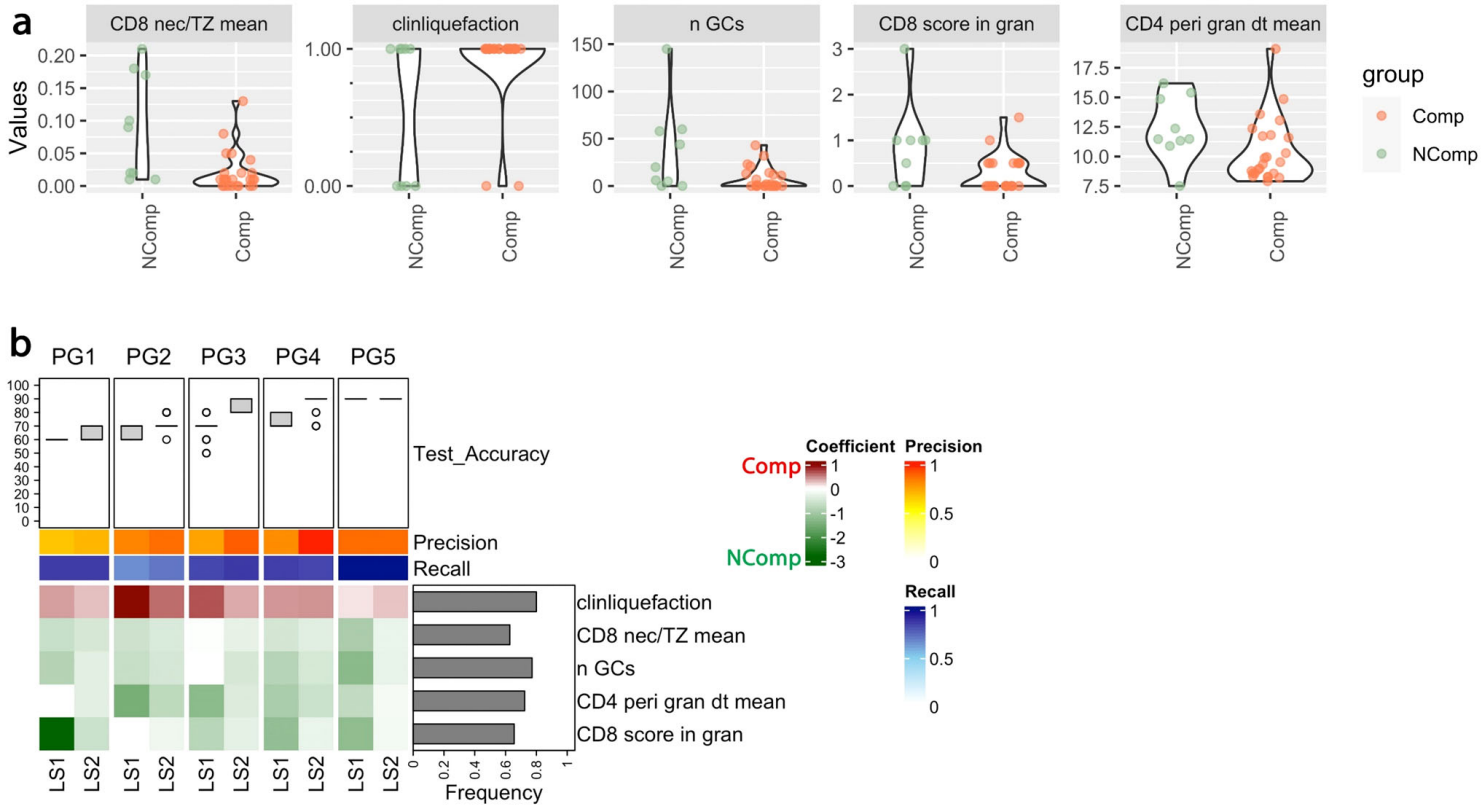
Patterns of CD8 infiltration, liquefaction and germinal centers correlate with the clinical course. **(a)** Pairwise analysis of depicted variables associated with uncomplicated (NComp, n=9) and complicated course (Comp, n=24) has been illustrated. The box plots on the top show the test accuracies of 50 models trained using k-fold cross validation (k=3) for each PG and learning steps (LS) and evaluated based on the test data. 70% of the samples (n = 23) together with the augmented ones (n = 11) were used for training and 30% of the samples (n = 10) were used for testing. The mean of the precision and recall of the models calculated based on the test data has been illustrated as two horizontal color bars. The bar plot at the right side presents the frequency of selection of the biomarkers. The heatmap shows the average value of the coefficients of the models. The green values indicate a positive, the red values a negative prognostic effect on the outcome "course". Unadjusted p-values (Wilcoxon-Mann-Whitney test) for comparisons: CD8 nec/TZ mean = 0.005, clinliquefaction = 0.020, n GCs = 0.043, CD8 score in gran = 0.050, and CD4 peri gran dt mean = 0.072. Correlation coefficient: CD8 nec/TZ mean= -0.55, clinliquefaction = 0.42, n GCs = -0.47, CD8 score in gran = -0.41, and CD4 perigran dt mean = -0.32. **(b)** Binomial logistic regression analysis with classifiers categorizing NComb and Comp for different partition groups (PGs). clinliquefaction = signs of liquefaction; CD8 nec/TZ mean = Ratio of CD8 T cell density (number per area) in the granuloma necrosis over the T cell zone; n GCs = number of germinal centers; CD8 score in gran = CD8 T cell density inside the granuloma necrosis, relative to the granuloma wall (IC 0: <0.1%, IC 1: 0.1-0.4%, IC 2: 0.5-0.9%, IC 3: 0.1-100%); CD4 peri gran dt mean = Delaunay: Mean distance in μm of CD4 positive T cells in the perigranulomatous area.

Classifiers categorizing NComp and Comp for different PGs, are displayed in **Figure 3**: Maintained lymphoid structures as well as higher numbers of germinal centers in the affected lymph node were associated with an uncomplicated course, which was also the case for a higher CD8+ T-cell ratio necrosis vs. T-zone, a higher score of CD8+ T-cells inside the granuloma necrosis and higher mean distances between perigranulomatous CD4+ T-cells. Liquefaction, as measured clinically by ultrasound or MRI, predicted a complicated course (**Figures 1c1-3, C8, C9, b6, b7, d2**, **Figure 3** for the underlying classification).

**Figure 4:**
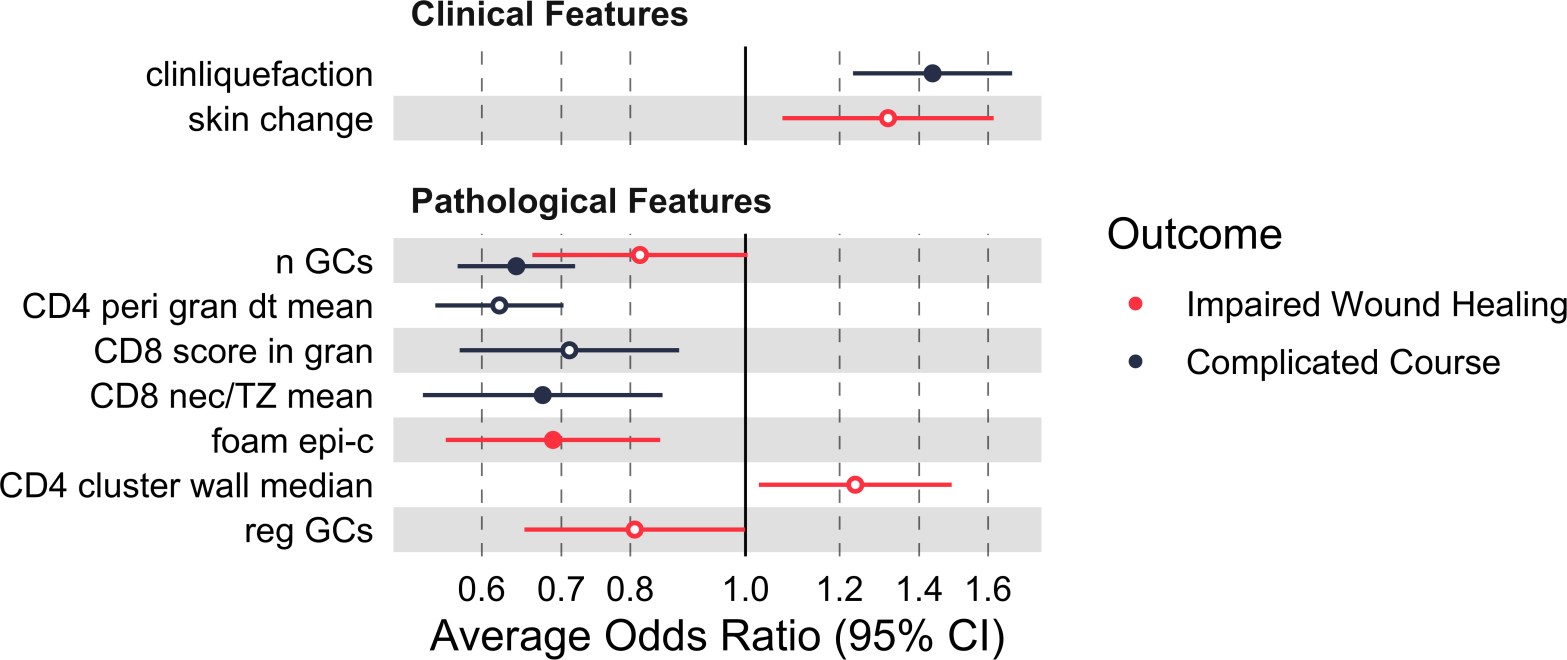
Clinical and histopathological features predicting outcome. Germinal center number, scattered distribution of granuloma associated CD4+ T-cells, higher CD8+ T-cell density inside the necrosis and foamy epitheloid cells were associated with a favorable outcome. Forest plot of the Average of Odds Ratio (OR) associated to each outcome (95% confidence interval (CI), binomial logistic regression models). Solid dots: significant variables. Hollow dots: non-significant ones (significance threshold at adjusted p < 0.05). Details of the ORs and related CIs are given in **Supplementary Table 2**. clinliquefaction = signs of liquefaction (MRI or ultrasound); skin change = clinical skin symptoms (e.g. discoloration); nGCs = number of germinal centers; CD4 peri gran dt mean = Delaunay: Mean distance between CD4+ T-cells in the perigranulomatous area; CD8 score in gran = CD8+ T-cell density (aim for necrosis and set positive cells in relation to the positive cells inside the granuloma wall: IC 0: <0.1%, IC 1: 0.1-0.4%, IC 2: 0.5-0.9%, IC 3: 1-100%); CD8 nec/TZ mean = ratio granuloma necrosis/T-cell zone: Mean numbers of CD8+ positive cells per mm^2^; foam epi-c = foam cell aspect: epitheloid cells; CD4 cluster wall median = median number of CD4+ T-cells forming a cluster inside the granuloma wall; reg GCs = regressive germinal centers.

The forest plot of the average odds ratios of the relevant variables is displayed in **Figure 4**. Visualizations of the distributions across the risk groups for the outcomes of wound healing and course are provided in the supplement (**Supplementary Figures 2**, **3**).

### Association of germinal center frequency and granuloma maturity with disease course

**Figure 5:**
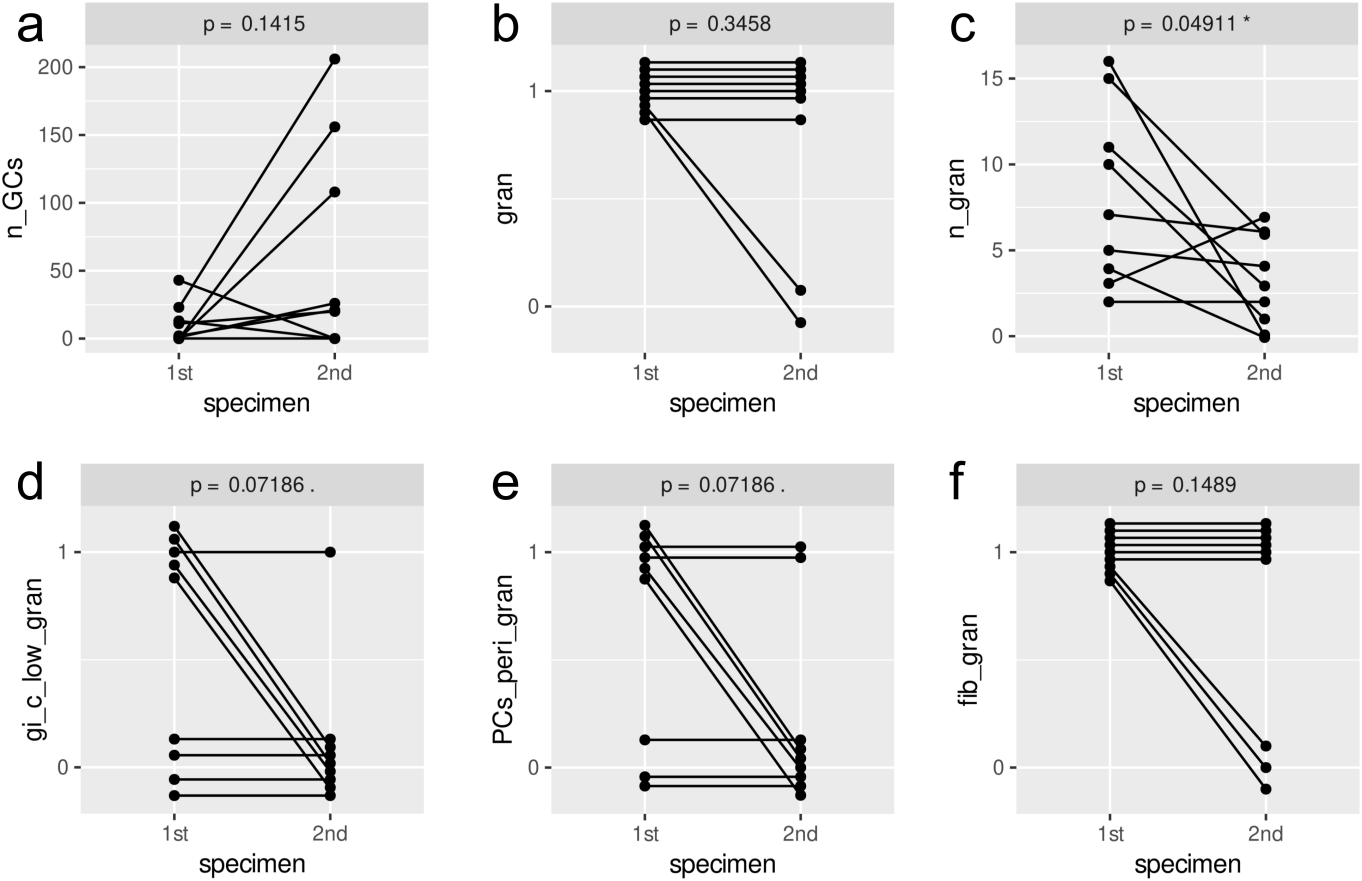
Intra-individual changes of selected histological parameters between different timepoints (p-values calculated by paired Wilcoxon signed-rank sum test). The parameters are as follows: **(a)** n_GCs = number of germinal centers; **(b)** gran = granulomas present (1) or absent (0); **(c)** n_gran = number of granulomas in 10x objective lens field of view; **(d)** gi_clow_gran = less than 10 giant cells per 2 HPF in hot spots; **(e)** PCs_peri_gran = granuloma-associated plasmacytosis; **(f)** fib_gran = perigranulomatous fibrosis.

In 9 patients, samples were collected at two time points each. The timing of the second operation depended on the clinical course, leading to variable intervals between biopsies. The mean duration between first and second surgery was 3 months (range < 1 month to 6 months, median of 3 months). While most of the intraindividual changes over time were not significant, some trends appear notable. In most of the second samples, the number of germinal centers was higher (**Figure 5a**). Yet, the number of granulomas (10x magnification) was significantly lower in the second sample compared to the first, granulomas being absent in the second sample in two cases (**Figures 5b, c**). Furthermore, whereas the granulomas showed less than 10 giant cells per 2 HPF in hot spots in the first sample, this was not the case in the second samples (**Figure 5d**). For foamy epitheloid cells and mean numbers of giant cell nuclei, no time-dependent pattern was found. In contrast, perigranulomatous plasmacytosis and fibrosis were observed less frequently in the second sample (**Figures 5e, f**). Patterns of CD8+ T cell distribution and density did not differ significantly between timepoints.

### Correlation of features independent of the disease course and wound healing

We previously found that expression of the nuclear markers p53 and nitrotyrosine was characteristic for mycobacteria-induced formation of granuloma-associated giant cells, both in mice and humans (9). To further dissect types of granulomas and giant cells, we combined these parameters with the immunohistochemistry of BCL2 as an antiapoptotic protein (17, 18) and BCL6, which counterbalances BCL2 in the lymphatic compartment and plays a role in osteoclastic giant cell formation (19, 20).

We found all samples to comprise nitrotyrosine-positive macrophages in granulomas (**Supplementary Figures 5E, G**). Whereas the minority of giant cells was BCL2-positive, the majority was positive for BCL6 and p53 (**Supplementary Figures 5A-D, F**). Histological features are exemplified in the **Supplementary Figure 5**.

The proportion of BCL2-positive giant cells correlated with the maximum number of giant cell nuclei, hyperplastic follicles, hyperplastic germinal centers, and was inversely associated with the minimal distance between CD4+ T-cells in the T-zone, the CD4/CD8 ratio in the granuloma wall and positively with the median CD8 density in the T-zone. Concerning BCL6, the proportion of positive giant cell nuclei correlated with the maximum distance between CD4+ T-cells in the T-zone. The proportion of p53-positive giant cells correlated with giant cell rich granulomas. A negative correlation was found for lower giant cell density in granulomas. Higher proportions of p53 nuclei in giant cells positively correlated with higher CD8+ T-cell densities in the granuloma necrosis and with suppurative necrosis. Beyond that, they showed the same correlations as the aforementioned proportion of p53 giant cells.

## Discussion

With their ability to cause long-lasting local invasive tissue inflammation in immunocompetent children, NTM infections require substantial medical attention. To date, histological analysis of affected lymph nodes mainly aims at confirming etiology, i.e., identifying granulomatous inflammation and detection of NTM by microscopy or molecular methods, and excluding alternative diagnoses including malignant lymphoma. Accordingly, the wealth of information potentially extracted from a complex tissue sample, which needs considerable effort to be collected (including general anesthesia) is usually not exploited. The investigation presented here was therefore based on the hypothesis that in-depth histologic analysis of surgical samples may reveal prognostic and thus therapeutically meaningful tissue response patterns. The insidious course inherent to NTM infections inevitably leads to a variability in disease stages when patients seek expert medical attention. This limits histopathological standardization of disease stages, despite the relative homogeneity of causative NTM species, i.e., *M. avium-intracellulare* complex in our cohort. The relatively low density of mycobacteria in the granulomas that we observed by Ziel-Neelsen staining is typical for NTM lymphadenitis (21). We also cannot exclude that lesions which resolve spontaneously or that resolve with antibiotics alone may have similar pathology that regresses. Another potential variable is the genetic predisposition of patients. Multifokal lymphadenitis is the most common finding in in the clinical syndrome called “Mendelian susceptibility to mycobacterial disease”, with 21 genes known so far being affected. NTM can be isolated in approximately a quarter of cases. However, isolated cervical lymphadenits that was the dominating clinical feature in our study, is relatively rare in this condition (22).

Maintained lymphoid structures, as easily identifiable germinal centers, were signs of lower tissue damage and uncomplicated courses in our cohort, the opposite did occur in complicated courses. This is in line with other studies describing the tissue damage based upon the extension of clinical involvement (23, 24).

Necrotizing granulomas, at least partly displaying caseous necrosis, were a constant feature in our and another cohort (23). Together with our findings of also fibrinoid, suppurative necrosis and mixed patterns, we speculate that the quality of the necrosis might be time- or host-dependent rather than being NTM-specific. Of note, there are morphological overlaps with tuberculosis, bartonella infection and listeriosis (25–28). The functional study of the latter group linked the morphology of suppurative granulomas to TNF-α- and IFN-γ-dependent indoleamine 2,3-dioxygenase induction, which is important for the clearance of the *L. monocytogenes* infection and on the other hand an important immunomodulator towards suppression, possibly preventing tissue damage (28, 29). We did not find significant correlations between suppurative necrosis and complications in our data, which might be due to the different pathogen or due to variable intervals between infection onset and surgical intervention. Another factor pointing in a similar direction are our previous findings that NO-dependent p53 inhibition, cholesterol and lipid accumulation strongly support formation of giant cells, which themselves are potential NO sources (8, 9, 30). In accordance with these findings, higher numbers of giant cell nuclei were associated with an uncomplicated course (**Supplementary Figure 3**, mean n nuclei gi-c) in our cohort. This further supports the hypothesis of higher NO levels in individual giant cells with consecutively higher killing capacity against mycobacteria. A lack in NO and thus an increase in p53 function may mediate granuloma contraction. On the other hand, NO may propagate giant cells as permissive hosts of intracellular mycobacteria (9) leading to chronic infection and tissue damage mediated by e.g., proteinases (31, 32). Accordingly, our patients with complicated courses and repeat surgery displayed lower rates of mature granuloma features in the second sample (**Figure 5**). Yet, mycobacterial persistence and chronic infection may be prevented by early and sustained T-cell help (33). Together with our data, we can postulate that the distribution pattern of T-cells predicts antimycobacterial activity. A more scattered distribution of single CD4+ T-cells or small groups inside the granuloma wall was associated with good wound healing, whereas larger clusters were not (**Figure 1d**, **Supplementary Figure 2**). An unexpected finding was that a higher density of CD8+ T-cells in the granuloma core was associated with good wound healing and an uncomplicated course of the disease (**Figure 1d**, **Supplementary Figures 2**, **3**). We do not think that CD8+ T-cells are directly involved in mycobacterial killing. However, CD8+ T-cells are known to be strong macrophage activators, e.g. in case of insufficient killing in hemophagocytic lymphohistiocytosis (34). Possibly, CD8+ T-cells are important to promote antimycobacterial granuloma activity. A potential, antigen-independent mechanism could be via TLR2-mediated innate production of IFN-γ by CD8+ T Cells, leading to the activation of macrophages (35). Of note, TLR2 is activated by mycobacteria, which we also used in a previous experimental setting (9). To verify antigen-dependency, it could be worth sequencing the T-cell receptor genes of microdissected T-cells to look for clonal enrichment. However, the causal involvement of CD8+ T cells remains speculative, and their presence could just as well be an epiphenomenon. Further work is needed to better understand the biology of granuloma formation and maturation in the context of NTM lymphadenitis. The data presented here may provide some starting points in this regard.

From a clinical perspective, the identification of patients at high risk of a complicated course may improve therapeutic stratification beyond our previously proposed clinical risk score (13). Impaired wound healing and complicated disease courses in NTM infections in children may be predicted from tissue samples by reduced lymphoid structures, lower giant cell nuclear counts, low CD8+ cell density inside the granuloma necrosis and CD4+ clustering inside the granuloma wall as indirect signs of an inadequate immune reaction to NTM. However, in order to identify robust correlations, an increased amount of clinical and histological data will be required.

Since the effect sizes of any single parameter identified here are rather modest, additional features or the combination of multiple parameters need to be explored for their potential to derive clinically meaningful predictions about prognosis. Despite the use of different partitions of training and test samples and multiple iterations of classifier training using a two-step learning process, there remains a risk of overfitting due to the small cohort size and the use of oversampling. Therefore, validating the results with an independent cohort seems important.

Ultimately, the development of automated algorithms integrating a limited set of prospective clinical data and quantifiable histological parameters, e.g., number of CD8+ T-cells, germinal centers and foamy epithelioid cells, could improve the clinical management of these patients.

## Data Availability

The original contributions presented in the study are included in the article/**Supplementary Material**. Further inquiries can be directed to the corresponding authors.
