# Supplementary material for: Immunopathogenic and clinical implications of advanced tissue analysis in non-tuberculous mycobacterial infections in children
Source: Front Immunol. 2025 Jun 26;16:1597074. doi: 10.3389/fimmu.2025.1597074 (PMC12257029; doi:10.3389/fimmu.2025.1597074)
Supplement: Supplementary file 1 [file SupplementaryFile1.docx]

**Supplemental Methods and Materials**

**Histology**

Histological evaluation was performed using digitized slides and a Leica DM2500 microscope (Leica, Wetzlar, Germany). Diameter of 10x field of view (FOV): 2.2 mm. Diameter of 40x FOV (HPF): 0.55 mm. Slide digitization was performed using a slide scanner with a 20x objective and a resolution of ~0.22 µm per pixel (Mirax Scan Pannoramic Scanner, 3DHistec, Hungary). For picture acquisition of scanned slides, the proprietary software (Pannoramic Viewer, version 1.15.4, 3DHistec) and the software QuPath (*1*) (version 0.5.0) were used. Abbreviations of the analysed items are provided in supplementary table 1. Histological analyses comprised qualitative and quantitative items. Qualitative items are e.g. regressive germinal centers (GCs), granulomas with sharply demarcated border, granuloma-associated plasma cell richness, foamy epitheloid cells, collagen fibres around granulomas, and lymphatic structure (if no residual lymph node could be identified). Quantitative items are e.g.: mean number of nuclei from 20 giant cells, number of GCs, number of granulomas in 10x FOV, maximum granuloma diameter in percent of 40x FOV, and maximum number of nuclei in 10 giant cells. BCL2, BCL6 and p53 stainings were evaluated in 10 giant cells. Representative giant cells were selected from 10 granulomas, or from the maximum number of granulomas present on the slide if less than 10 were observed. CD4 and CD8 positive T-cells were quantified in the granuloma wall, inside the necrosis, the perigranulomatous area and the T-cell zone using QuPath on the digitized whole slide scans of the sole or first tissue sample. Granuloma wall, necrosis and T-cell zone were annotated. For the perigranulomatous area, an 80 µm zone around the granuloma was chosen. The T-cell zone was labelled in the paracortex of the lymph node. If no maintained lymph node architecture was identifiable, lymphocytic infiltrates outside the granulomas were labelled as T-cell zone. For the quantification of the CD4 T-cells, cells were first identified using the cell detection tool in QuPath. Afterwards, a Random Forest classifier was trained to identify the CD4 T-cells, separated from macrophage populations as e.g. epithelioid and giant cells and CD4-negative lymphocytes. CD4 T-cells were evaluated according to their number per area and the distance between the cells (Delaunay analysis). Clusters were defined as cells of the same class connected by a line without interruption of cells belonging to another class (e.g. CD4+ macrophages). For CD8 positive T-cells, the positive cell detection tool was used and the cells were evaluated according to their number per area. Settings of the cell detection parameters were saved for every single slide section and are provided in the supplement. Moreover, CD8 positive T-cells were scored by two independent pathologists (MS and CN) in analogy to the immune cell score used in breast cancer (*2*). Briefly, the percentage of granuloma associated CD8 positive T-cells is semiquantitatively weighed against the granuloma wall: IC 0: <1%, IC 1: 1-4%, IC 2: 5-9%, IC 3: 10-100%, relative to granuloma wall area. CD8 positive T-cells inside the granuloma necrosis were evaluated separately, using one tenth of the score: IC 0: <0.1%, IC 1: 0.1-0.4%, IC 2: 0.5-0.9%, IC 3: 0.1-100%, relative to granuloma wall area. The mean value of both observers was used for calculations.

**Statistical evaluation**

**Relevant variables in prognosis and imputation**

The relevance of the variables to each outcome has been evaluated based on the correlation analysis of the variables to each outcome (abs(r) >0.3) and Wilcoxon-Mann-Whitney test (unadjusted p-value <0.1). The latter test has been done by partitioning the samples based on the two groups within each outcome. The R package of “stats” has been used for both of the tests. These relatively liberal cutoffs were selected to minimize the risk of overlooking any potential relevant variables for the outcomes “wound healing” and “course”.

After identifying relevant variables to each outcome, missing values were imputed. For imputation, the union of all of the relevant variables was split into two groups of variables with and without missing values. The latter group was called “reference variables” in our text, and K-nearest neighbor’s method was used for imputing missing values. The underlying assumption of using K-nearest neighbor’s method posits that the closest neighbors of a sample with missing data within the feature space of the reference variables are the most appropriate candidates for imputation. The package of “impute” in R with K=3 (*3*) has been used for imputation. Variables that have more than three missing values were not used for imputation and consequently for further analysis. Supplementary Table 3 presents relevant variables with missing values and associated sampleIDs, which were imputed in our study:

| **Variables** | **Sample IDs** | **No. NAs Missing Values** |
| --- | --- | --- |
| CD4/CD8 nec mean | 25; 26; 18 | 3 |
| n nuclei p53 gi-c | 8 | 1 |
| n nuclei 10 gi-c | 8 | 1 |
| n gran p53 gi-c | 8; 20 | 2 |
| clinliquefaction | 24; 11; 33 | 3 |

*Suppl table 1: Relevant variables, which missing values were imputed, with sample IDs.*

The p-value of the Wilcoxon-Mann-Whitney test as well as the correlation coefficient of each variable to the outcome were re-calculated after imputation.

Considering Wilcoxon-Mann-Whitney test and correlation analysis of the relevant variables, there is a subset of variables with a higher prognostic role among all of the relevant ones for each outcome (unadjusted p-value < 0.05). It seems important to highlight that all of the relevant variables affect the precision of our analysis due to the limited sample size and imbalanced categories. For this reason, if a significance filter was applied, certain relevant variables with p-values ≥ 0.05 might be erroneously identified as false negatives (*4*–*7*), and the use of multiple corrections can further increase the risk that important variables are incorrectly excluded from the analysis (*5*).

The adjusted p-values were calculated using the Benjamini-Hochberg correction. None of them were statistically significant enough to explain the differences between the two classes of each outcome alone, due to the small and imbalanced dataset. Supplementary Figure 3 illustrates the distinction between the unadjusted and adjusted p-values. The Supplementary Table, spreadsheet “SignificanceRelevantVariables,” provides detailed information on the p-values and adjusted ones for each variable, outcome, and test.

Therefore, we relied solely on the unadjusted p-values with a moderate threshold from the Wilcoxon-Mann-Whitney test and correlation analysis to identify a subset of relevant variables for use in the classification process. We aimed to determine whether these variables, when considered together, could contribute to distinguishing between the outcome classes robustly.

**Classification using BLR**

The classifiers were trained to predict the binary labels associated with each outcome, where the labels corresponded to the risk categories. To minimize bias from a particular split of the data, the training process was repeated across 5 different partition groups (PGs) of training and test samples. Within each PG and for each label of the outcome, the data was split into 70% for training and 30% percent for testing. To ensure balanced representation of risk categories during the learning process, we augmented the training data with a random subset of samples from minority groups for each outcome.

To train binary classifiers, Binomial Logistic Regression (BLR) was applied in two sequential learning steps. In the first learning step (LS1), the most important variables were recognized by training BLR models based on LASSO (*8*) repeated 50 times for each PG of each outcome. The top 5 frequently selected variables of each outcome were used to train robust classifiers in learning step 2 (LS2), where training was done using BLR and Ridge (*8*) 50 times for each PG of an outcome. The performance of binary classifiers of LS1 and LS2 was assessed using the respective test sets from PGs. Alongside test accuracies, precision and recall metrics were calculated for the test data. Additionally, Odds Ratios (OR) of the estimated coefficients of the ensemble of classifiers of LS2 were included.

Definitions regarding the terms and phrases used for this process are given as follows:

Labels: The categories associated to each outcome in our study, which are Comp vs NComp for complicated course of the disease and IWH vs GWH in the case wound healing. The binary classifiers are trained to recognize the correct label of a given sample for each outcome.

Train and Test samples: For a training process, the samples were partitioned into train and test sets. The parameters of the classifier were estimated using the training data. The estimated classifier was evaluated using test data to provide the test error, which is a measurement regarding the performance of the estimated classifier based on the instances it has never seen before (*9*). In this analysis, the percentages of train and test samples were 70% and 30% for each label of each outcome.

Partition Group: To avoid the bias of using a special partition of train and test samples, 5 independent partition groups (PG1-PG5) of train and test data were used for each outcome.

Augmented Samples: For the training set of each partition group to generate a balanced number of labels for each outcome, oversampling was done for the minor label of each outcome (*10*). In oversampling, a subset of training samples was selected randomly from the minor group (n = 11 for NComp; n = 2 for IWH) to be augmented to the training data. Supplementary Table 3 presents the number of samples of train and test as well as the number of augmented samples of each outcome.

Cross Validation: In order to have less bias to the training data and higher generalization ability of the estimated classifier regarding unseen data, k-fold cross validation (k=3 in our analysis) has been used for each training process (*11*). Using this technique, samples used for training were divided into k subsets or folds. The binary classifier is trained and evaluated k times, using one of the folds which was not used for training each time. The average of the error of this evaluation of each fold presents the model's generalization performance.

Learning Step: For each partition group of training (including augmented samples) and test data, two Learning Steps based on three-fold cross validation were applied. In the first learning step (LS1), the most important variables were recognized by training BLR models using LASSO (*8*). The training process was repeated 50 times, and the test error of classification was calculated for each training process and for each partition group. Afterward, the top 5 frequently selected variables of LS1 were used to train robust BLR classifiers based on Ridge (*8*) in the second learning step (LS2). The learning process was done iteratively 50 times for each partition group, and test errors were calculated accordingly. The BLR models were trained using “glmnet” package in R (*8*,*12*).

Ensemble of classifiers: For each outcome and learning step, the training process was repeated 50 times for each partition group resulting in an ensemble of 250 classifiers.

Odds Ratio (OR): Considering the regularization term in the cost function of BLR models of glmnet, we have used the same code given in (*12*) to calculate the standard error of the estimated coefficients of a trained model. Each model’s OR and related confidence interval (CI) have been calculated based on the classifier’s coefficients and the associated standard error (*13*,*14*). Since an ensemble of classifiers was provided at LS2, the mean ORs of the BLR classifiers and related confidence interval (CI) were depicted in the forest plot for each outcome. In the supplementary table 2, all of the details regarding odds ratios, CIs, and related significance (Benjamini-Hochberg corrected) of all of the estimated models are given.

Precision and Recall: In addition to the test accuracies, the metrics of precision and recall were also computed using TP/(TP+FP) and TP/(TP+FN), respectively, where TP (True Positives), FP (False Positives), and FN (False Negatives) were evaluated based on the test data (*15*).

| **Wound healing** | | | | | | |
| --- | --- | --- | --- | --- | --- | --- |
| feature | | OR | CI_95 | p_val | adj. p_val | Significance |
| foam epi-c | | 0.6887356 | (0.559583032904986, 0.84769676729513) | 0.03740161 | 0,04331087 | * |
| n GCs | | 0.81512788 | (0.661744987180865, 1.00406270119059) | 0.09885149 | 0,13669693 |  |
| skin change | | 1.31806023 | (1.07415938788205, 1.61734171058718) | 0.06248485 | 0,07733855 |  |
| CD4 cluster wall median | | 1.23701621 | (1.02628726838062, 1.49101440331357) | 0.07160384 | 0,09219643 |  |
| reg GCs | | 0.80683521 | (0.651624819512367, 0.999015132620072) | 0.09349608 | 0,12744341 |  |
|  | |  |  |  |  |  |
|  | **Course of Disease** | | | | | |
| feature | | OR | CI_95 | p_val | adj. p_val | Significance |
| clinliquefaction | | 1.43679924 | (1.23157694184679, 1.67621849951768) | 0.02956143 | 0,03306604 | * |
| n GCs | | 0.64150633 | (0.572514994571794, 0.71881151330945) | 0.02047713 | 0,0218558 | * |
| CD4 peri gran dt mean | | 0.62079635 | (0.548290539955461, 0.702890301221885) | 0.05367818 | 0,06012066 |  |
| CD8 score in gran | | 0.7109679 | (0.574805369378999, 0.879385225742284) | 0.05668856 | 0,06998199 |  |
| CD8 nec/TZ mean | | 0.67513298 | (0.535276762058991, 0.851530604084271) | 0.04103556 | 0,04895804 | * |

*Suppl table 2: Odds ratios, confidence intervals, and related significance of all estimated models of the suppl. methods.*

**Supplemental Results**

|  | **This cohort** | **Original NTMkids study** | **p (Fisher's exact test, except for age (Wilcoxon))** |
| --- | --- | --- | --- |
| **Age at diagnosis, months (median)** | 32 | 28 | 0.6143603 |
| **Female, %** | 67 | 61 | 0.543468 |
| **Local skin symptoms, %** | 55 | 59 | 0.68486 |
| **General condition impaired, %** | 22 | 17 | 0.4387176 |
| **Affected region head/neck, %** | 91 | 96 | 0.3569574 |
| **Max lymph node size >2 cm, %** | 87 | 83 | 0.5886974 |
| **Liquefication of affected lymph node on US or MRI, %** | 79 | 70 | 0.2472098 |
| **Calcification of affected lymph node on US or MRI, %** | 5 | 23 | 0.06541914 |
| **Bilateral involvement of lymph nodes, %** | 7 | 9 | 1 |
| **Confluent lymph nodes, %** | 48 | 48 | 1 |
| **MAI complex of NTM identified, %** | 78 | 84 | 0.5110256 |
| **Complicated course, %** | 72 | 65 | 0.1946722 |
| **Impaired wound healing, %** | 44 | 43 | 1 |
| **Facial nerve palsy, %** | 22 | 7 | 0.001507844 |

*Suppl. table 3: Comparison between the histologically characterized cohort and the cohort of the original NTMkids study* (*16*)*.*

|  | n | mean | std | min | 25% | 50% | 75% | max |
| --- | --- | --- | --- | --- | --- | --- | --- | --- |
| foam epi-c | 33 | 0.33 | 0.48 | 0 | 0 | 0 | 1 | 1 |
| n GCs | 33 | 15.76 | 28.9 | 0 | 0 | 2 | 20 | 145 |
| skin change | 33 | 0.58 | 0.5 | 0 | 0 | 1 | 1 | 1 |
| reg GCs | 33 | 0.42 | 0.5 | 0 | 0 | 0 | 1 | 1 |
| clinliquefaction | 30 | 0.8 | 0.41 | 0 | 1 | 1 | 1 | 1 |
| CD4 cluster wall median | 33 | 3.82 | 3.95 | 1 | 2 | 2 | 4 | 20 |
| CD4 peri gran dt mean (µm) | 33 | 10.96 | 2.79 | 7.5 | 8.58 | 10.28 | 12.36 | 19.1 |
| CD8 score in gran | 33 | 0.42 | 0.63 | 0 | 0 | 0 | 0.5 | 3 |
| CD8 nec/TZ mean | 33 | 0.04 | 0.06 | 0 | 0.005 | 0.01 | 0.05 | 0.21 |

*Suppl. table 4: Distribution of relevant variables. Foamy epithelioid cells (foam epi-c), skin change and clinical signs of liquefaction (clinliquefaction) are dichotomous values. Note that clinliquefaction was available for 30 patients, which was the reason to impute 3 missing values for further calculations. The further abbreviation names are given as follows: n GCs = number of germinal centers; skin change = skin change; reg GCs = regressive germinal centers; CD4 cluster wall median = median number of CD4+ T-cells forming a cluster inside the granuloma wall; CD4 peri gran dt mean = Delaunay: Mean distance (µm) between CD4+ T-cells in the perigranulomatous area); CD8 score in gran = density of CD8+ T-cells in the granuloma necrosis, relative to the granuloma wall: IC 0: <0.1%, IC 1: 0.1-0.4%, IC 2: 0.5-0.9%, IC 3: 1-100%; CD8 nec/TZ mean = Ratio of CD8 T cell density (number per mm^2^) in the granuloma necrosis over the T cell zone.*

**Supplemental References**

1. Bankhead P, Loughrey MB, Fernández JA, Dombrowski Y, McArt DG, Dunne PD, McQuaid S, Gray RT, Murray LJ, Coleman HG, James JA, Salto-Tellez M, Hamilton PW. QuPath: Open source software for digital pathology image analysis. Sci Rep. 2017 Dec 4;7(1):16878–16878.

2. Schildhaus H-U. Der prädiktive Wert der PD-L1-Diagnostik. Pathol. 2018 Oct 26;39(6):498–519.

3. Troyanskaya O, Cantor M, Sherlock G, Brown P, Hastie T, Tibshirani R, Botstein D, Altman RB. Missing value estimation methods for DNA microarrays. Bioinformatics. 2001 Jun 1;17(6):520–5.

4. Jenkins DG, Quintana-Ascencio PF. A solution to minimum sample size for regressions. PloS One. 2020 Feb 21;15(2):e0229345–e0229345.

5. Kaler AS, Purcell LC. Estimation of a significance threshold for genome-wide association studies. BMC Genomics. 2019 Jul 29;20(1):618–618.

6. Lo SK, Li IT, Tsou TS, See L. [Non-significant in univariate but significant in multivariate analysis: a discussion with examples]. Chang Yi Xue Za Zhi. 1995 Jun;18(2):95–101.

7. Saccenti E, Hoefsloot HCJ, Smilde AK, Westerhuis JA, Hendriks MMWB. Reflections on univariate and multivariate analysis of metabolomics data. Metabolomics. 2013 Oct 26;10(3):361–74.

8. Friedman J, Hastie T, Tibshirani R. Regularization Paths for Generalized Linear Models via Coordinate Descent. J Stat Softw [Internet]. 2010;33(1). Available from: http://dx.doi.org/10.18637/jss.v033.i01

9. Géron A. Hands-on machine learning with Scikit-Learn, Keras, and TensorFlow: concepts, tools, and techniques to build intelligent systems. Third edition. Beijing Boston Farnham Sebastopol Tokyo: O’Reilly; 2023. 834 p. (Data science / machine learning).

10. He H, Ma Y, editors. Imbalanced Learning: Foundations, Algorithms, and Applications [Internet]. 1st ed. Wiley; 2013 [cited 2025 Feb 19]. Available from: https://onlinelibrary.wiley.com/doi/book/10.1002/9781118646106

11. James G, Witten D, Hastie T, Tibshirani R. An Introduction to Statistical Learning: with Applications in R [Internet]. New York, NY: Springer US; 2021 [cited 2025 Feb 19]. (Springer Texts in Statistics). Available from: https://link.springer.com/10.1007/978-1-0716-1418-1

12. Standard Errors in GLMNET : r/statistics [Internet]. [cited 2024 Mar 10]. Available from: https://www.reddit.com/r/statistics/comments/1vg8k0/standard_errors_in_glmnet/?rdt=64781.%202014

13. Starbuck C. Logistic Regression. In: The Fundamentals of People Analytics [Internet]. Cham: Springer International Publishing; 2023 [cited 2025 Feb 19]. p. 223–38. Available from: https://link.springer.com/10.1007/978-3-031-28674-2_12

14. Moore DS, McCabe GP, Craig BA. Introduction to the practice of statistics. Ninth edition. New York: W.H. Freeman, Macmillan Learning; 2017. 1 p.

15. Powers DMW. Evaluation: from precision, recall and F-measure to ROC, informedness, markedness and correlation. 2020 [cited 2025 Feb 19]; Available from: https://arxiv.org/abs/2010.16061

16. Kuntz M, Kohlfürst DS, Feiterna-Sperling C, Krüger R, Baumann U, Buchtala L, Elling R, Grote V, Hübner J, Hufnagel M, Kaiser-Labusch P, Liese J, Otto E-M, Rose MA, Schneider C, Schuster V, Seidl M, Sommerburg O, Vogel M, von Bernuth H, Weiß M, Zimmermann T, Nieters A, Zenz W, Henneke P, NTMkids Consortium. Risk Factors for Complicated Lymphadenitis Caused by Nontuberculous Mycobacteria in Children. Emerg Infect Dis. 2020 Mar;26(3):579–86.
