# Supplementary material for: Immunopathogenic and clinical implications of advanced tissue analysis in non-tuberculous mycobacterial infections in children
Source: Front Immunol. 2025 Jun 26;16:1597074. doi: 10.3389/fimmu.2025.1597074 (PMC12257029; doi:10.3389/fimmu.2025.1597074)
Supplement: Supplementary file 2 [file SupplementaryFile2.pptx]

## Slide 1
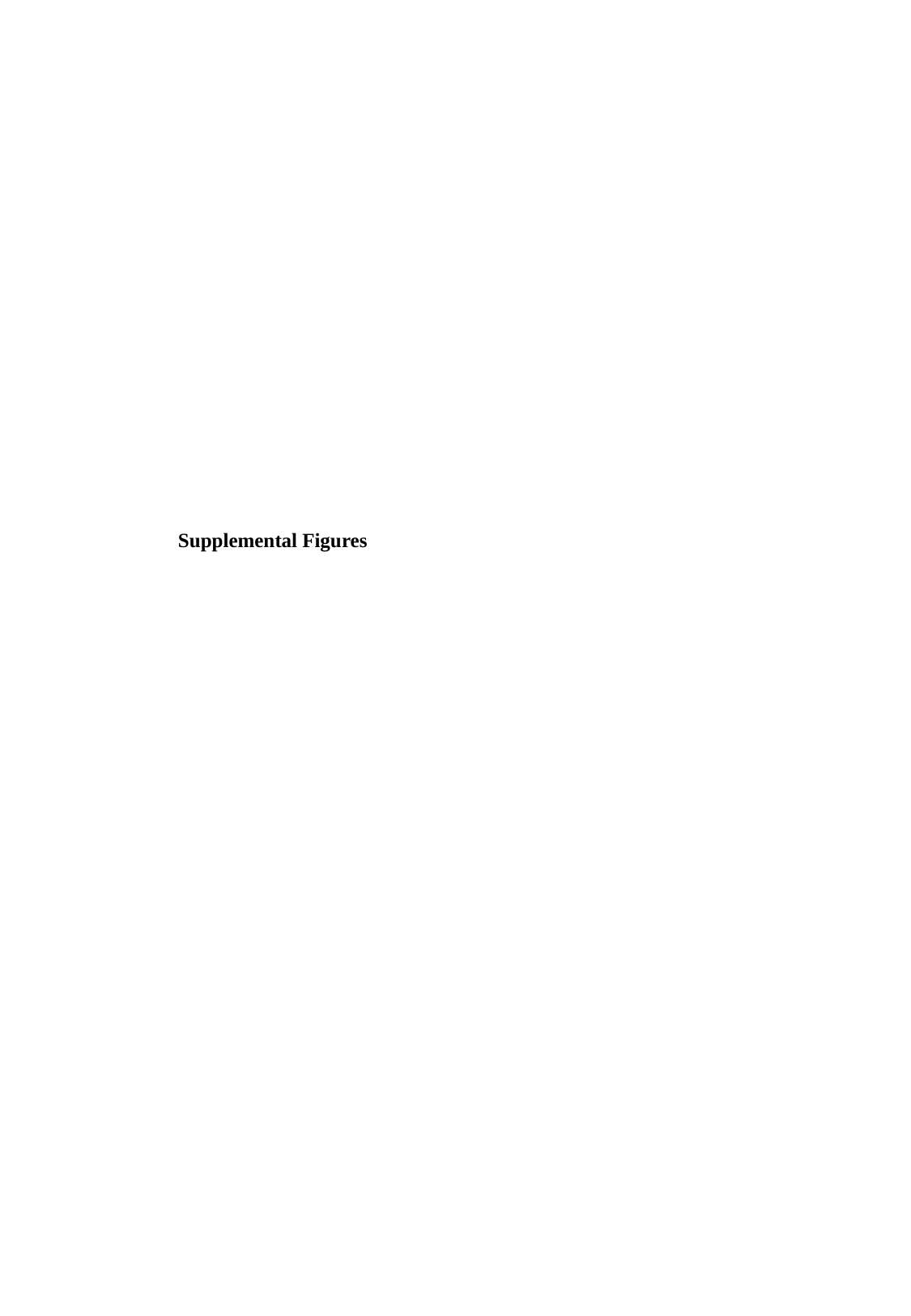

Supplemental Figures

## Slide 2
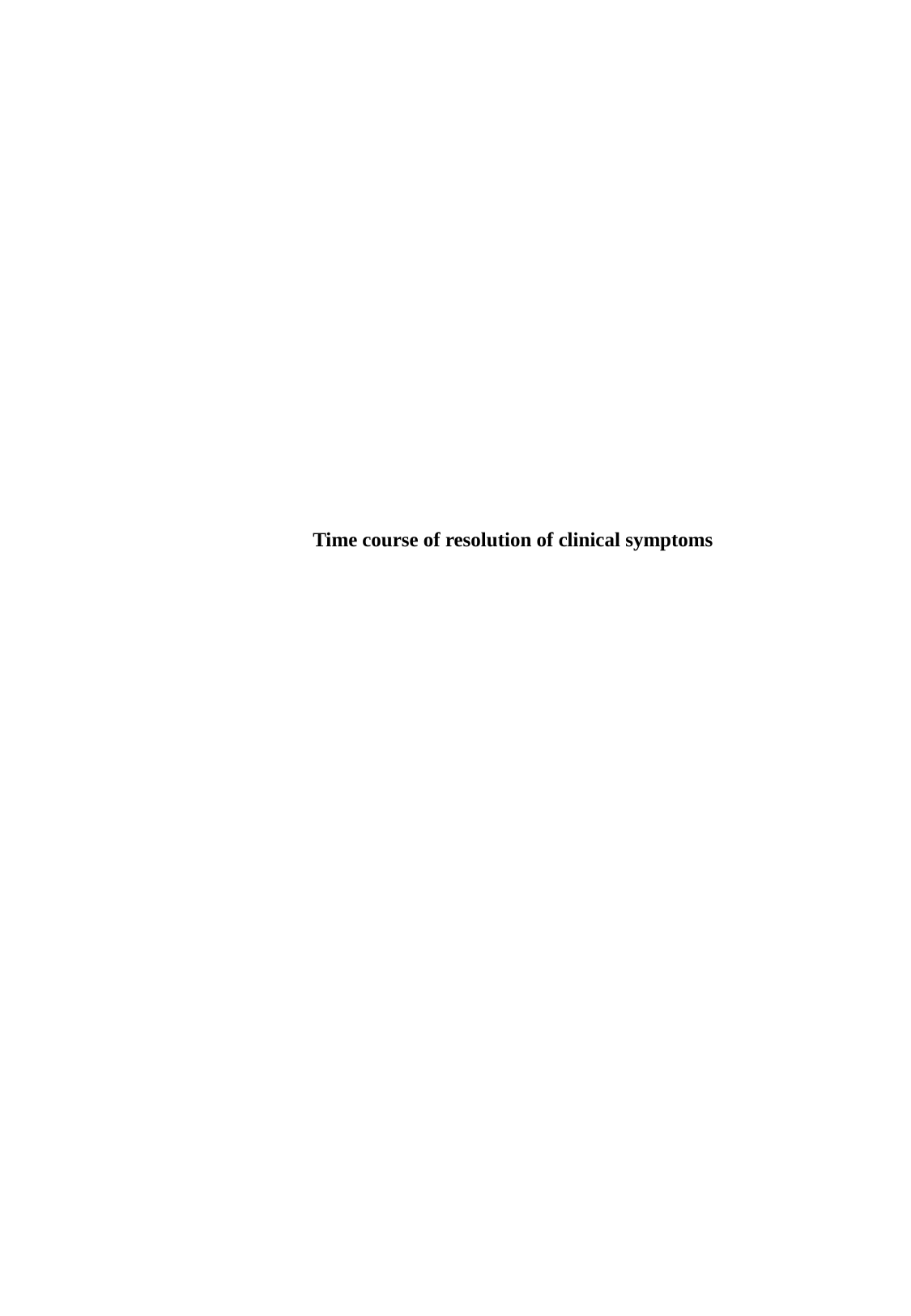

Time course of resolution of clinical symptoms

## Slide 3
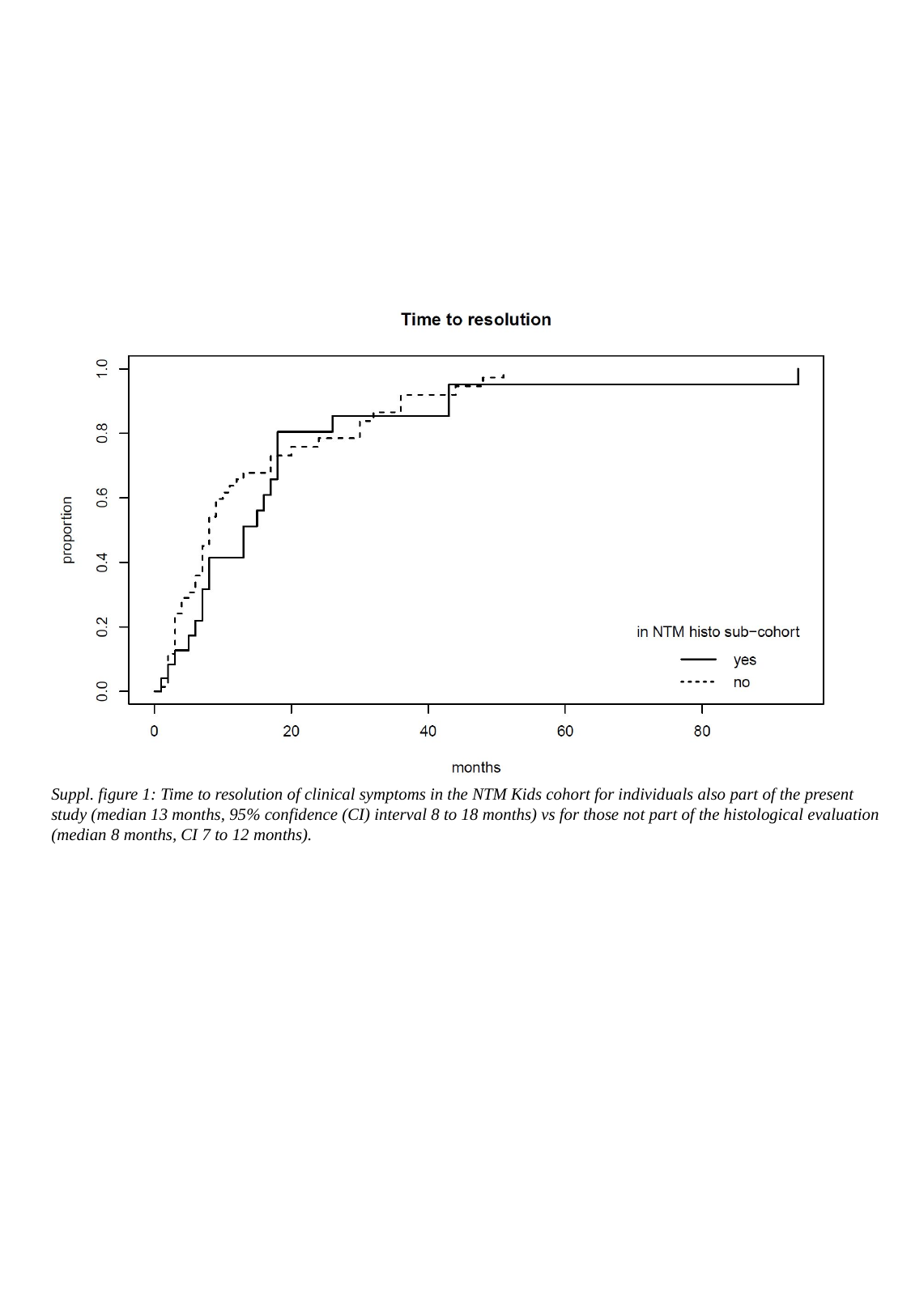

Suppl. figure 1: Time to resolution of clinical symptoms in the NTM Kids cohort for individuals also part of the present study (median 13 months, 95% confidence (CI) interval 8 to 18 months) vs for those not part of the histological evaluation (median 8 months, CI 7 to 12 months).

## Slide 4
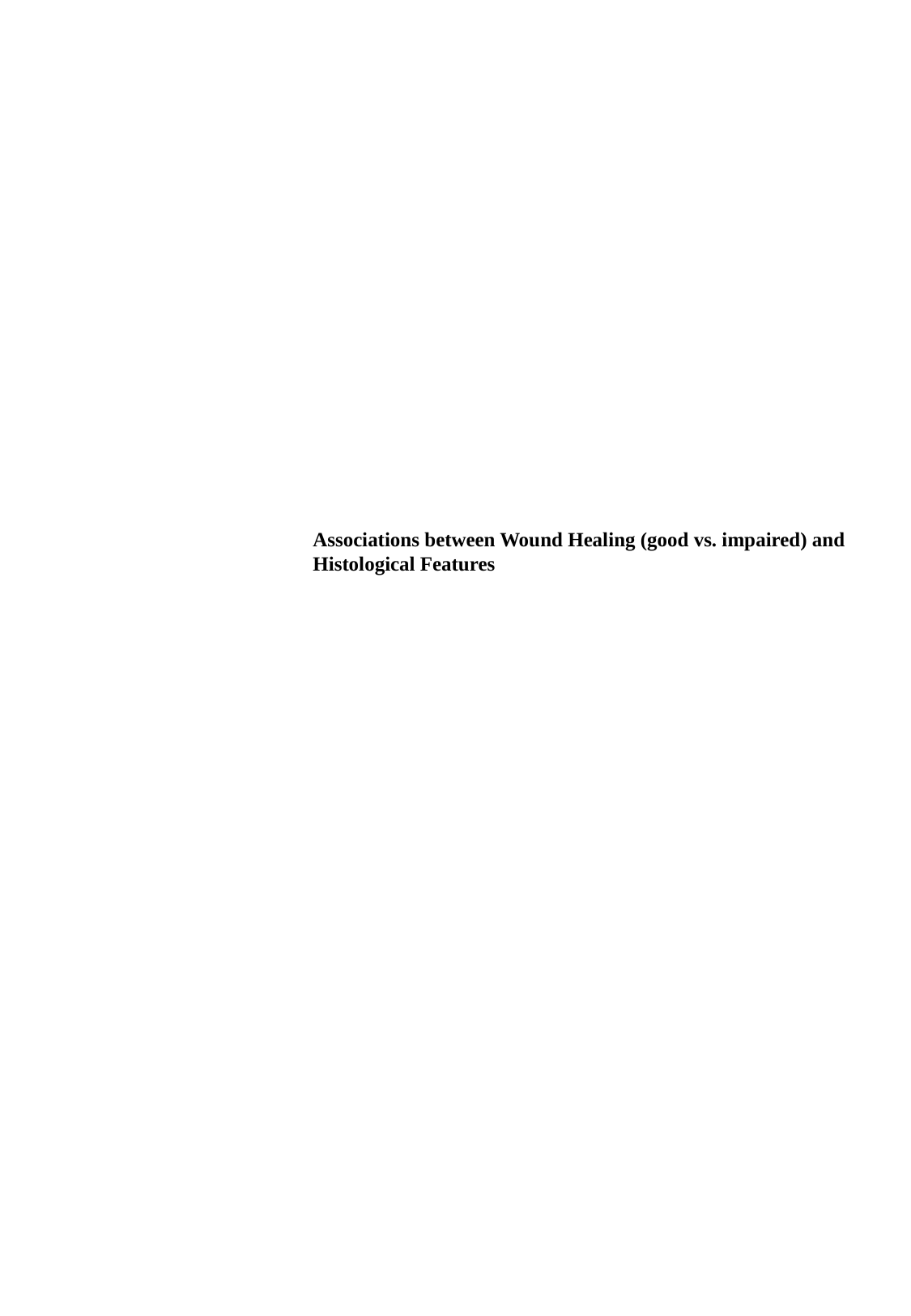

Associations between Wound Healing (good vs. impaired) and Histological Features

## Slide 5
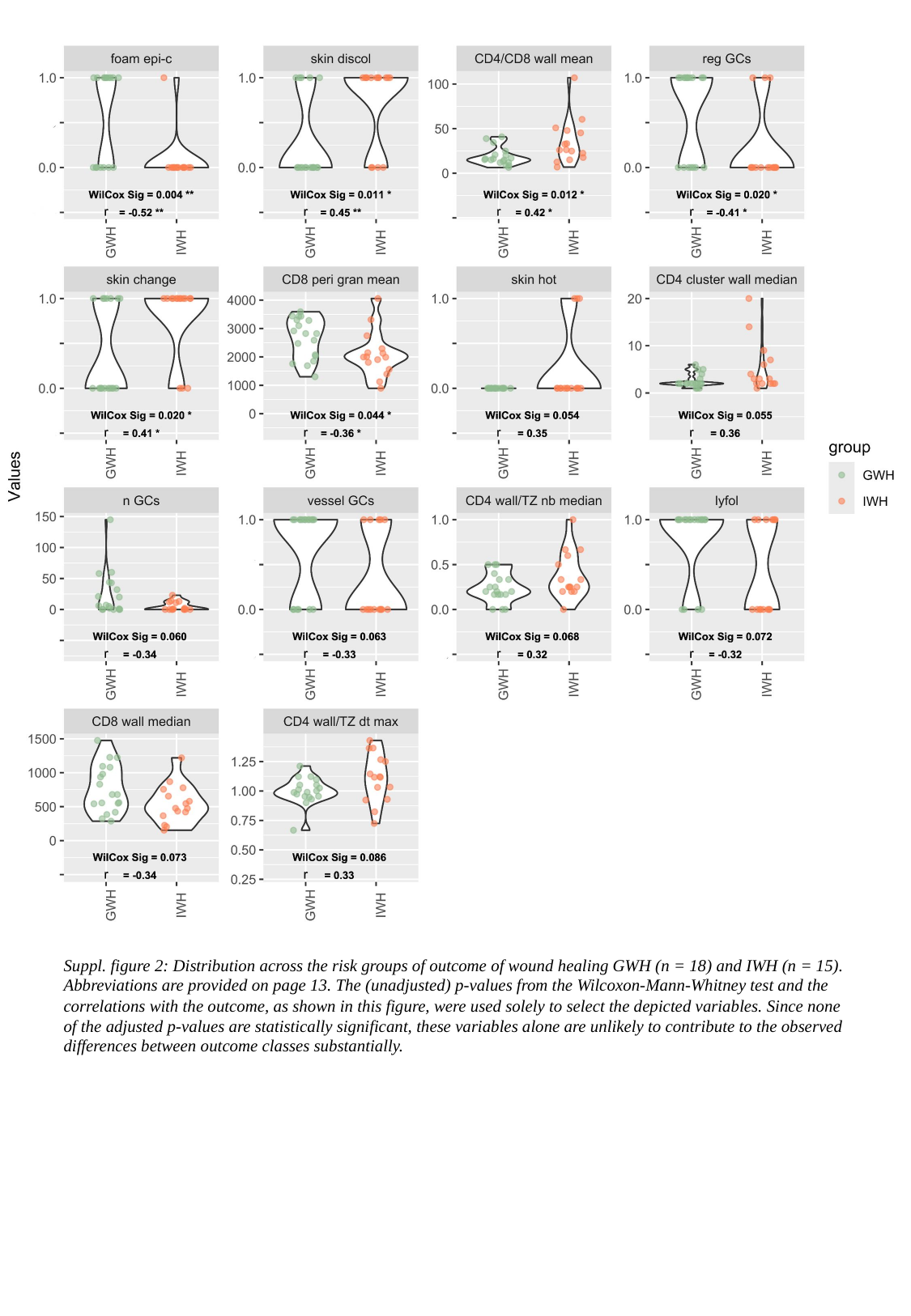

Suppl. figure 2: Distribution across the risk groups of outcome of wound healing GWH (n = 18) and IWH (n = 15). Abbreviations are provided on page 13. The (unadjusted) p-values from the Wilcoxon-Mann-Whitney test and the correlations with the outcome, as shown in this figure, were used solely to select the depicted variables. Since none of the adjusted p-values are statistically significant, these variables alone are unlikely to contribute to the observed differences between outcome classes substantially.

## Slide 6
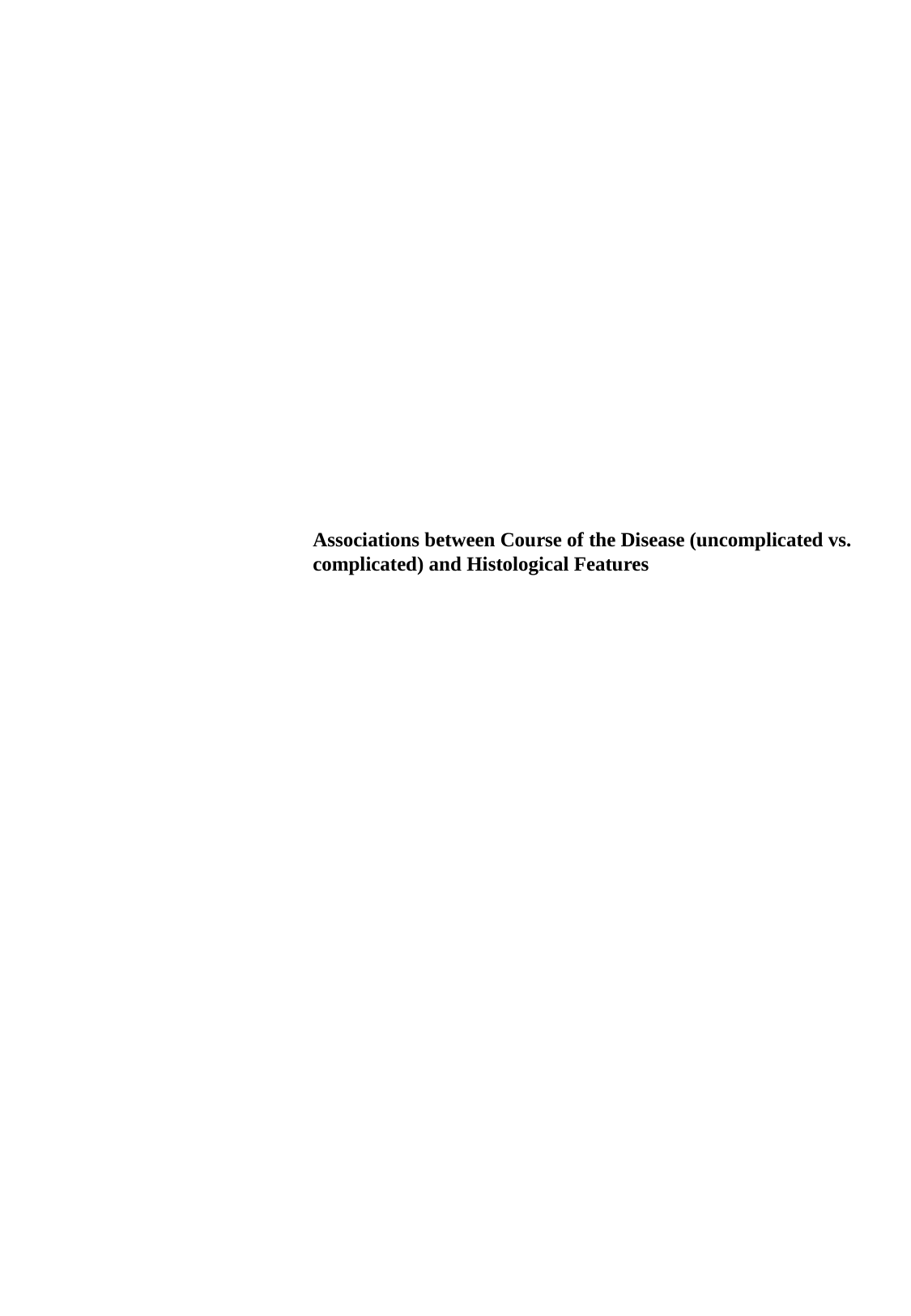

Associations between Course of the Disease (uncomplicated vs. complicated) and Histological Features

## Slide 7
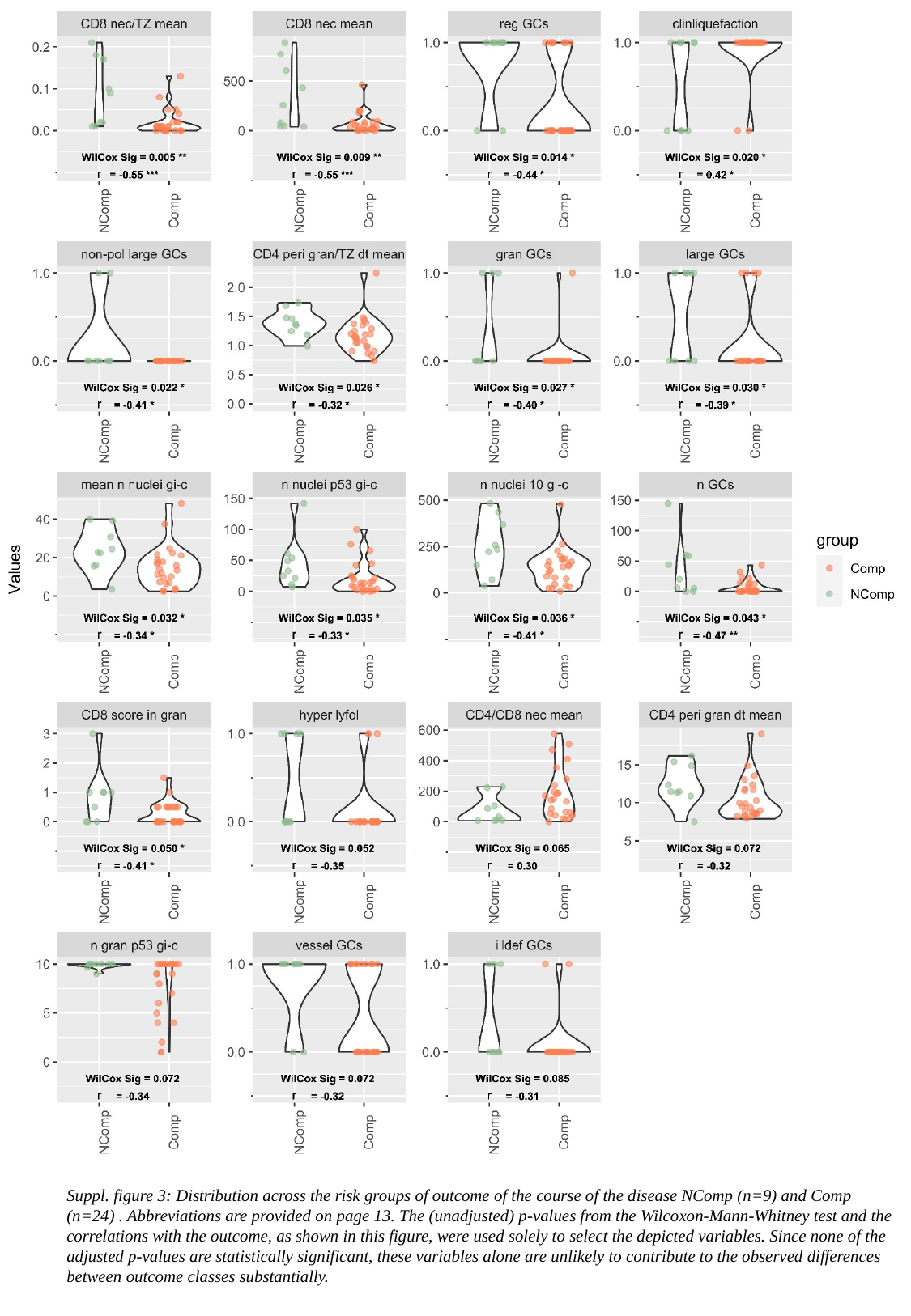

Suppl. figure 3: Distribution across the risk groups of outcome of the course of the disease NComp (n=9) and Comp (n=24) . Abbreviations are provided on page 13. The (unadjusted) p-values from the Wilcoxon-Mann-Whitney test and the correlations with the outcome, as shown in this figure, were used solely to select the depicted variables. Since none of the adjusted p-values are statistically significant, these variables alone are unlikely to contribute to the observed differences between outcome classes substantially.

## Slide 8
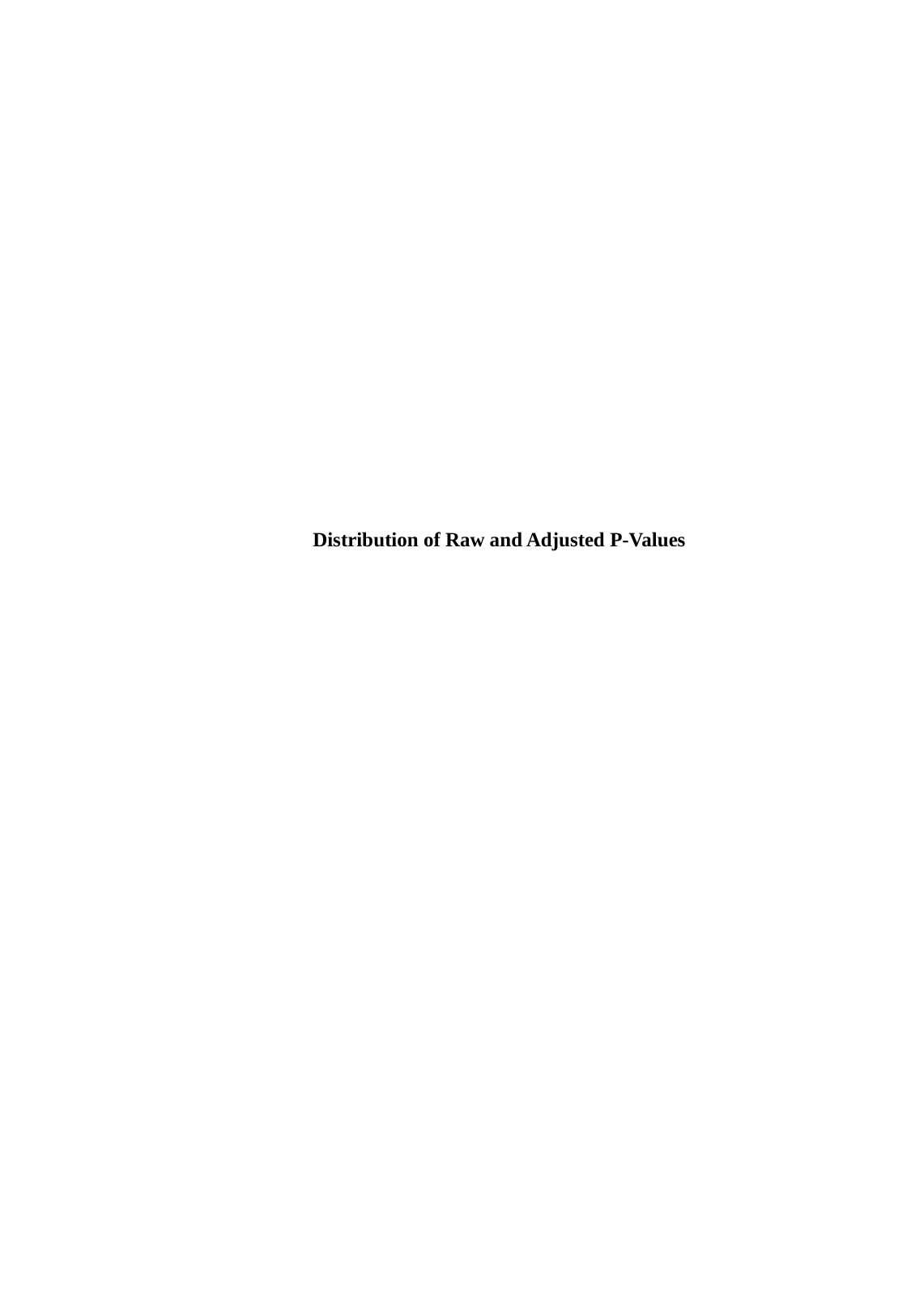

Distribution of Raw and Adjusted P-Values

## Slide 9
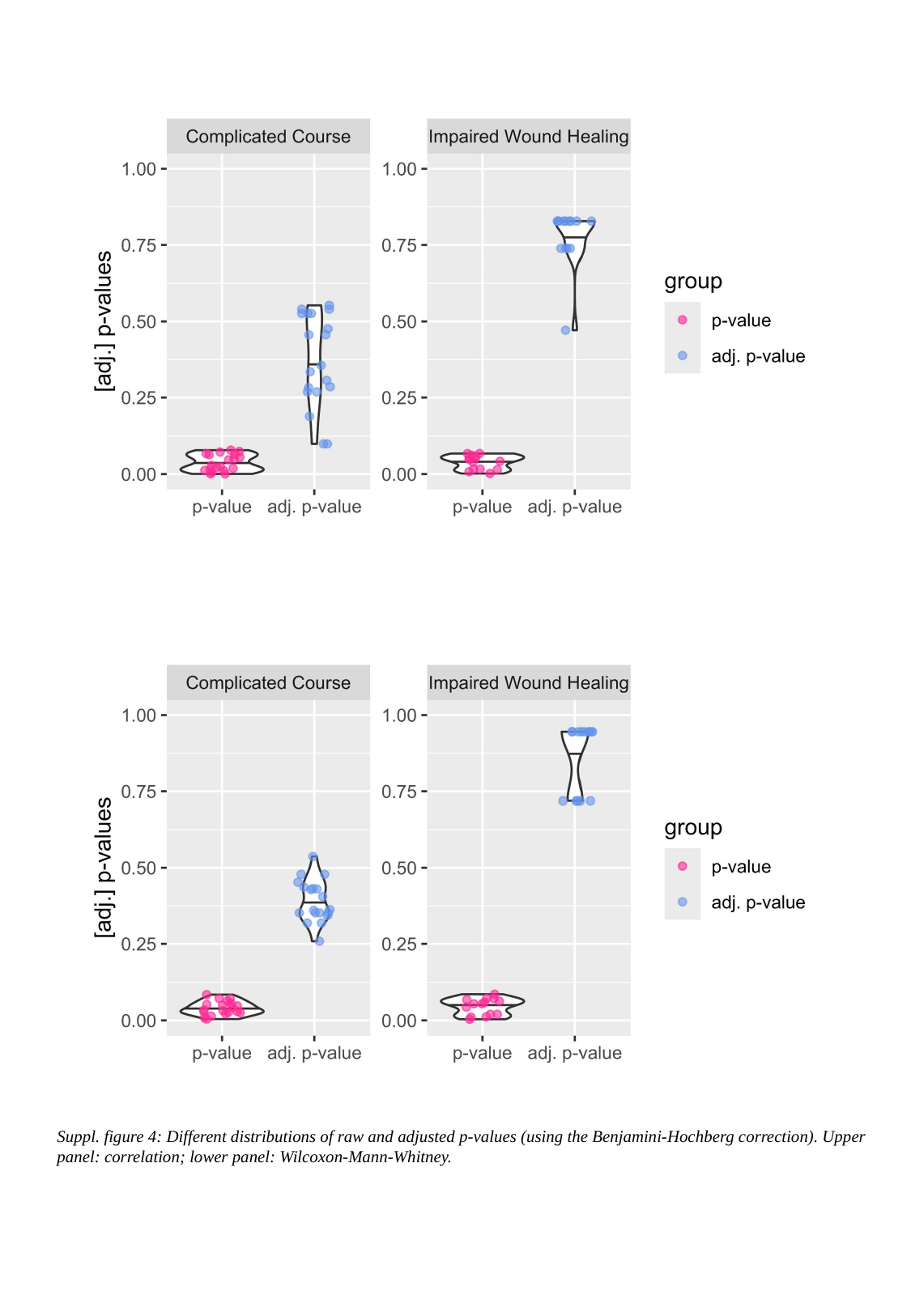

Suppl. figure 4: Different distributions of raw and adjusted p-values (using the Benjamini-Hochberg correction). Upper panel: correlation; lower panel: Wilcoxon-Mann-Whitney.

## Slide 10
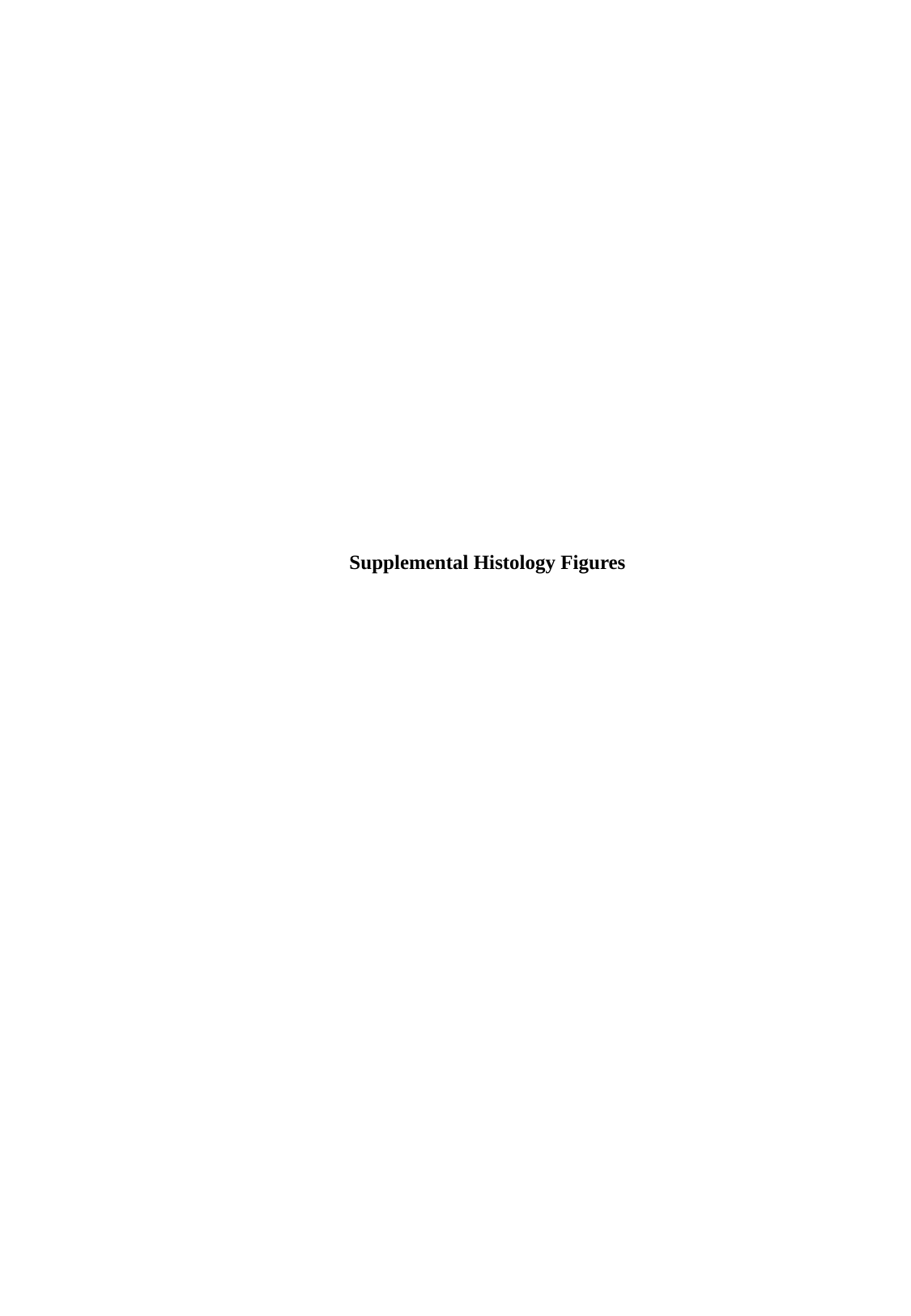

Supplemental Histology Figures

## Slide 11
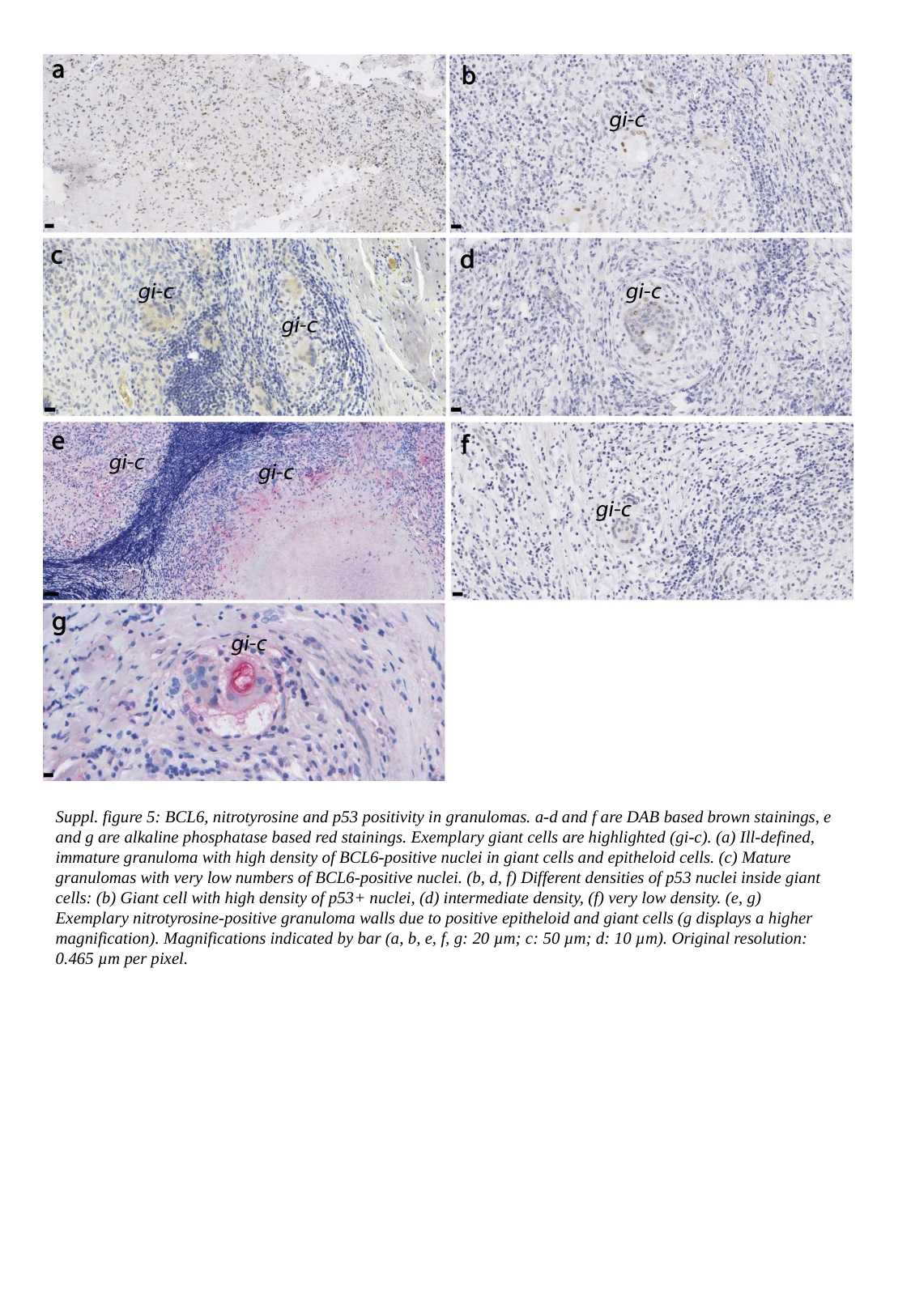

Suppl. figure 5: BCL6, nitrotyrosine and p53 positivity in granulomas. a-d and f are DAB based brown stainings, e and g are alkaline phosphatase based red stainings. Exemplary giant cells are highlighted (gi-c). (a) Ill-defined, immature granuloma with high density of BCL6-positive nuclei in giant cells and epitheloid cells. (c) Mature granulomas with very low numbers of BCL6-positive nuclei. (b, d, f) Different densities of p53 nuclei inside giant cells: (b) Giant cell with high density of p53+ nuclei, (d) intermediate density, (f) very low density. (e, g) Exemplary nitrotyrosine-positive granuloma walls due to positive epitheloid and giant cells (g displays a higher magnification). Magnifications indicated by bar (a, b, e, f, g: 20 µm; c: 50 µm; d: 10 µm). Original resolution: 0.465 µm per pixel.

## Slide 12
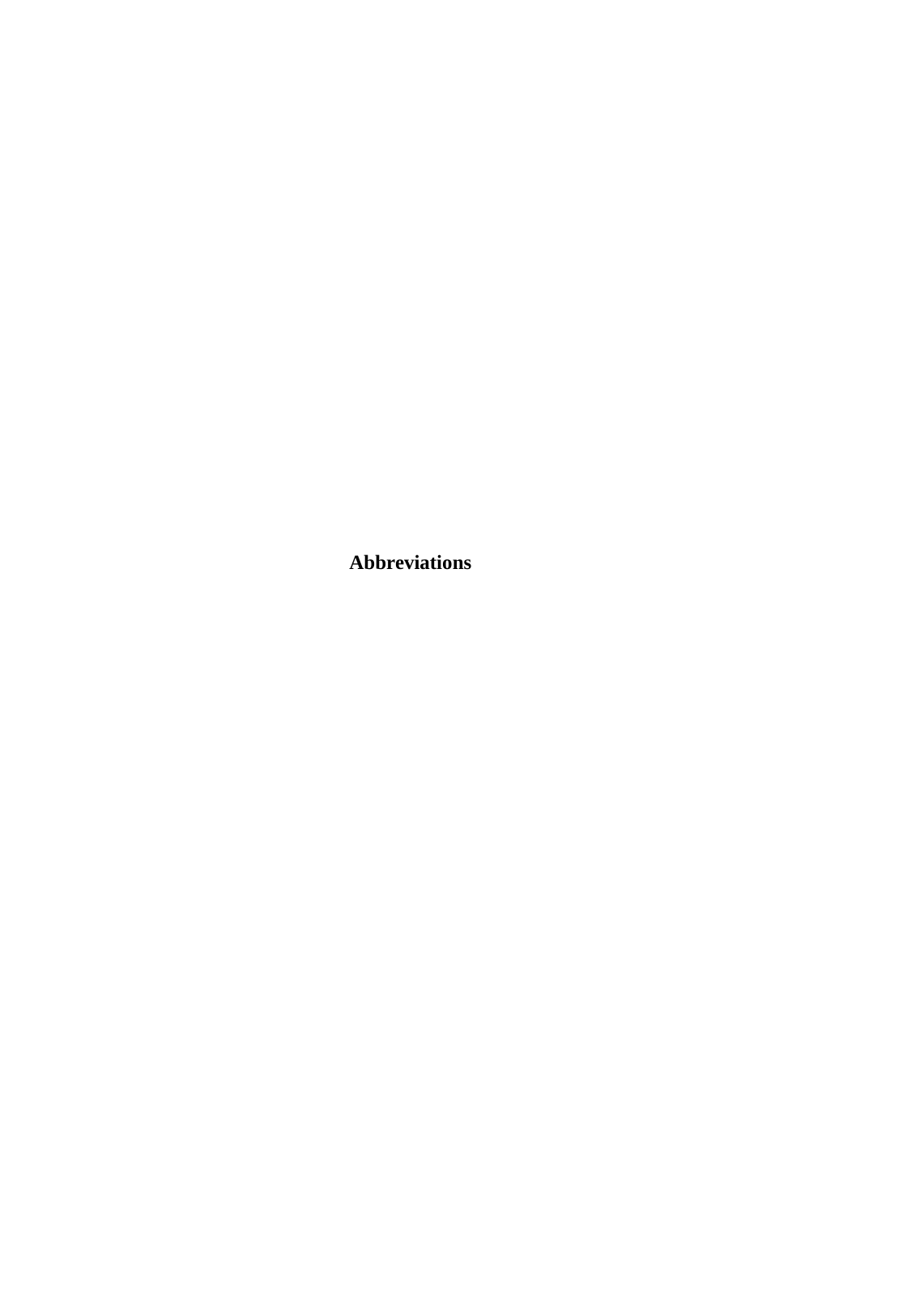

Abbreviations

## Slide 13
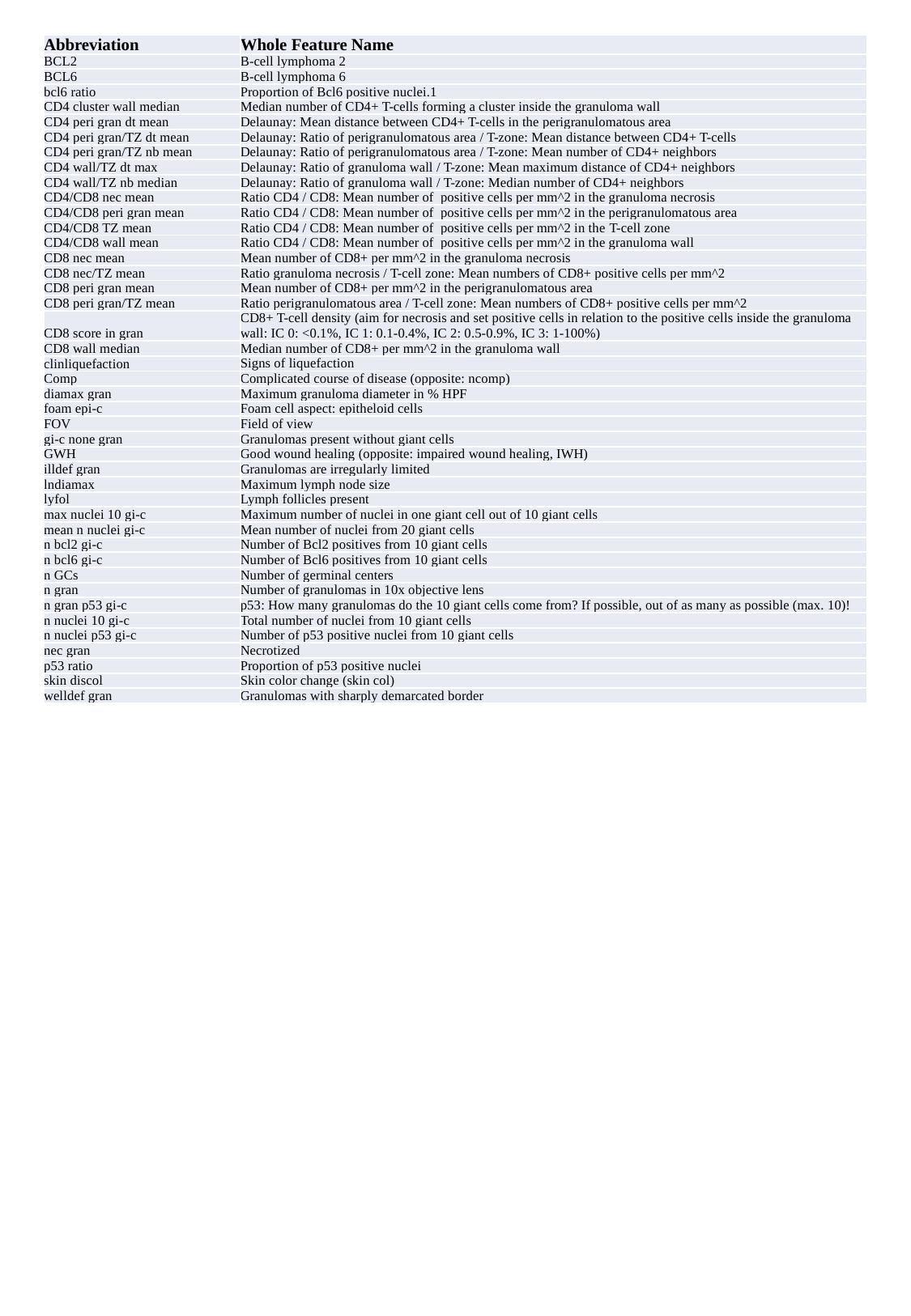

| Abbreviation | Whole Feature Name |
| --- | --- |
| BCL2 | B-cell lymphoma 2 |
| BCL6 | B-cell lymphoma 6 |
| bcl6 ratio | Proportion of Bcl6 positive nuclei.1 |
| CD4 cluster wall median | Median number of CD4+ T-cells forming a cluster inside the granuloma wall |
| CD4 peri gran dt mean | Delaunay: Mean distance between CD4+ T-cells in the perigranulomatous area |
| CD4 peri gran/TZ dt mean | Delaunay: Ratio of perigranulomatous area / T-zone: Mean distance between CD4+ T-cells |
| CD4 peri gran/TZ nb mean | Delaunay: Ratio of perigranulomatous area / T-zone: Mean number of CD4+ neighbors |
| CD4 wall/TZ dt max | Delaunay: Ratio of granuloma wall / T-zone: Mean maximum distance of CD4+ neighbors |
| CD4 wall/TZ nb median | Delaunay: Ratio of granuloma wall / T-zone: Median number of CD4+ neighbors |
| CD4/CD8 nec mean | Ratio CD4 / CD8: Mean number of positive cells per mm^2 in the granuloma necrosis |
| CD4/CD8 peri gran mean | Ratio CD4 / CD8: Mean number of positive cells per mm^2 in the perigranulomatous area |
| CD4/CD8 TZ mean | Ratio CD4 / CD8: Mean number of positive cells per mm^2 in the T-cell zone |
| CD4/CD8 wall mean | Ratio CD4 / CD8: Mean number of positive cells per mm^2 in the granuloma wall |
| CD8 nec mean | Mean number of CD8+ per mm^2 in the granuloma necrosis |
| CD8 nec/TZ mean | Ratio granuloma necrosis / T-cell zone: Mean numbers of CD8+ positive cells per mm^2 |
| CD8 peri gran mean | Mean number of CD8+ per mm^2 in the perigranulomatous area |
| CD8 peri gran/TZ mean | Ratio perigranulomatous area / T-cell zone: Mean numbers of CD8+ positive cells per mm^2 |
| CD8 score in gran | CD8+ T-cell density (aim for necrosis and set positive cells in relation to the positive cells inside the granuloma wall: IC 0: <0.1%, IC 1: 0.1-0.4%, IC 2: 0.5-0.9%, IC 3: 1-100%) |
| CD8 wall median | Median number of CD8+ per mm^2 in the granuloma wall |
| clinliquefaction | Signs of liquefaction |
| Comp | Complicated course of disease (opposite: ncomp) |
| diamax gran | Maximum granuloma diameter in % HPF |
| foam epi-c | Foam cell aspect: epitheloid cells |
| FOV | Field of view |
| gi-c none gran | Granulomas present without giant cells |
| GWH | Good wound healing (opposite: impaired wound healing, IWH) |
| illdef gran | Granulomas are irregularly limited |
| lndiamax | Maximum lymph node size |
| lyfol | Lymph follicles present |
| max nuclei 10 gi-c | Maximum number of nuclei in one giant cell out of 10 giant cells |
| mean n nuclei gi-c | Mean number of nuclei from 20 giant cells |
| n bcl2 gi-c | Number of Bcl2 positives from 10 giant cells |
| n bcl6 gi-c | Number of Bcl6 positives from 10 giant cells |
| n GCs | Number of germinal centers |
| n gran | Number of granulomas in 10x objective lens |
| n gran p53 gi-c | p53: How many granulomas do the 10 giant cells come from? If possible, out of as many as possible (max. 10)! |
| n nuclei 10 gi-c | Total number of nuclei from 10 giant cells |
| n nuclei p53 gi-c | Number of p53 positive nuclei from 10 giant cells |
| nec gran | Necrotized |
| p53 ratio | Proportion of p53 positive nuclei |
| skin discol | Skin color change (skin col) |
| welldef gran | Granulomas with sharply demarcated border |
